# Chiral probes for α_1_-AGP reporting by species-specific induced circularly polarised luminescence†
†Electronic supplementary information (ESI) available. See DOI: 10.1039/c8sc00482j


**DOI:** 10.1039/c8sc00482j

**Published:** 2018-02-19

**Authors:** Sergey Shuvaev, Elizaveta A. Suturina, Kevin Mason, David Parker

**Affiliations:** a Department of Chemistry , Durham University , South Road , Durham , DH1 3LE , UK . Email: david.parker@dur.ac.uk; b School of Chemistry , The University of Southampton , Highfield , Southampton SO17 1BJ , UK

## Abstract

Luminescence spectroscopy has been used to monitor the selective and reversible binding of pH sensitive, macrocyclic lanthanide complexes, **[LnL^1^]**, to the serum protein α_1_-AGP, whose concentration can vary significantly in response to inflammatory processes. On binding α_1_-AGP, a very strong induced circularly-polarised europium luminescence signal was observed that was of opposite sign for human and bovine variants of α_1_-AGP – reflecting the differences in the chiral environment of their drug-binding pockets. A mixture of **[EuL^1^]** and **[TbL^1^]** complexes allowed the ratiometric monitoring of α_1_-AGP levels in serum. Moreover, competitive displacement of **[EuL^1^]** from the protein by certain prescription drugs could be monitored, allowing the determination of drug binding constants. Reversible binding of the sulphonamide arm as a function of pH, led to a change of the coordination environment around the lanthanide ion, from twisted square antiprism (TSAP) to a square antiprismatic geometry (SAP), signalled by emission spectral changes and verified by detailed computations and the fitting of NMR pseudocontact shift data in the sulphonamide bound TSAP structure for the Dy and Eu examples. Such analyses allowed a full definition of the magnetic susceptibility tensor for **[DyL^1^]**.

## Abstract 2

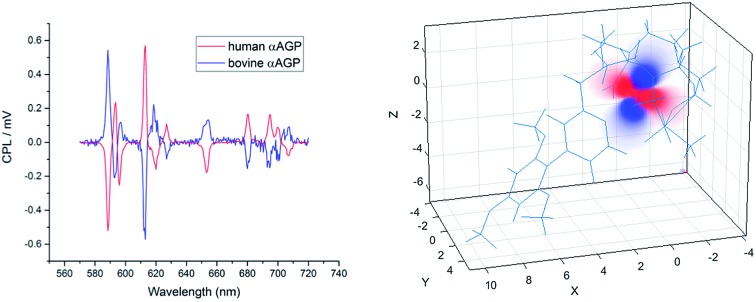
Luminescence spectroscopy has been used to monitor the selective and reversible binding of pH sensitive, macrocyclic lanthanide complexes, **[LnL^1^]**, to the serum protein α_1_-AGP, whose concentration can vary significantly in response to inflammatory processes.

## Introduction

The acute phase proteins found in human serum play a multitude of roles *in vivo*. Deviations from their normal concentration values can indicate the onset or presence of certain diseases. The second most abundant protein is α_1_-acid glycoprotein, α_1_-AGP, and fluctuations in its serum concentration merit close attention.^1^,^2^ Indeed, it has been hailed as the most important of four key circulating biomarkers that can be used to assess the five-year risk of mortality by any cause.^3^ It has been shown that α_1_-AGP transports a wide range of basic drugs in human plasma, *e.g.* methadone, disopyramide and many common anaesthetics such as lidocaine and bupivacaine, often competing with the more abundant serum albumin in this regard.^4^–^6^ Up to a 4-fold increase in its concentration is observed as a consequence of different inflammatory processes, including severe chronic diseases, such as cancer and HIV.^7^ This significant change of the concentration in response to external stimulus, characteristic for the acute phase proteins, inevitably affects drug metabolism and bioavailability, necessitating revised drug prescription for patients with inflammation.^8^,^9^

**Fig. 1:**
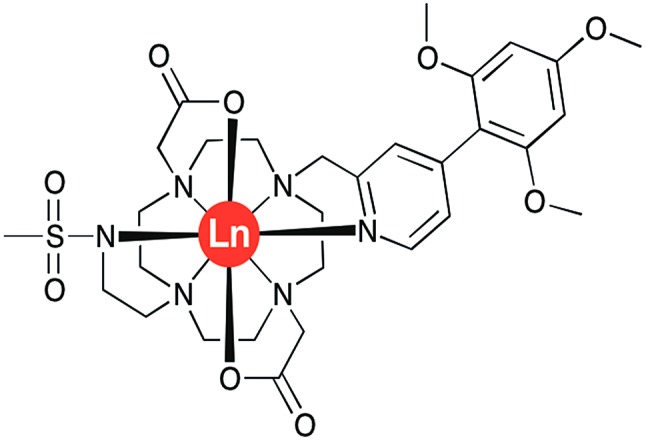
Molecular structure of **[LnL^1^]**, Ln = Y, Eu, Tb and Dy.

**Fig. 2:**
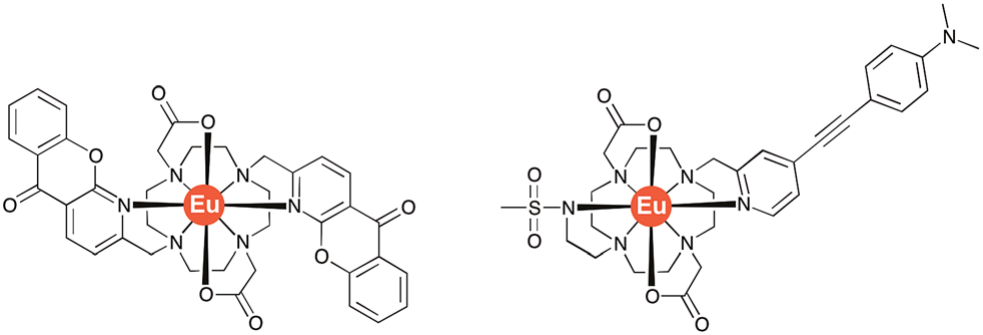
Molecular structures of the bis-azaxanthone complex, **[EuL^2^]**^12^ (left) and of **[EuL^3^]**^13^ (right).

However, there are a very limited number of publications on α_1_-AGP selective probes, with even fewer reporting their performance in serum. In the present paper, we report a series of new pH-responsive lanthanide complexes **[LnL^1^]** (Ln = Y, Eu, Tb, Dy; Fig. 1), which show a pronounced and species-dependent affinity towards α_1_-AGP, and a remarkable variation in metal complex helicity in the protein bound form, that is a function of AGP type and of local pH. The conjugated chromophore has a strong ICT transition that imparts sensitivity to changes in its local environment. Earlier, we reported a europium DO2A-based probe, **[EuL^2^]**, bearing two azaxanthone chromophores that showed selective binding for both α_1_-AGP and α_1_-antitrypsin (α_1_-AAT) with respect to human serum albumin (Fig. 2).^10^–^12^ It showed an increase of the total emission intensity and a large induced circularly polarised luminescence (CPL) upon binding these proteins only. It was hypothesised that the europium complex was embedded within the drug-binding pocket, and was bound to the carboxylate oxygen atom of a Glu-64 residue of α_1_-AGP, following dissociation of one of the azaxanthone nitrogen atoms. Juxtaposition of the amino-acid sequences in α_1_-AGP for different mammalian species reveals considerable discrepancies in the residues lining the drug-binding pocket, suggesting variable drug-binding affinities for different species. However, systematic analysis of the impact of these deviations on drug binding affinity is limited.

## Results and discussion

### Synthetic procedures

The complexes **[LnL^1^]** (Ln = Y, Eu, Tb, Dy) were synthesised by successive alkylation reactions of a DO2A-ethyl ester precursor (Scheme S1†). The chromophore was synthesised *via* a Suzuki–Miyaura cross-coupling reaction in a microwave reactor, using a Pd(TTP)_2_Cl_2_ catalyst. The freshly prepared benzylic methanesulphonate derivative of this chromophore was reacted with DO2A-ethyl ester, followed by reverse-phase HPLC purification. The isolated tri-substituted cyclen derivative was subsequently reacted with *N*-methanesulfonyl-aziridine, and RP-HPLC purification yielded the diethyl ester of ligand L^1^. The ester was hydrolysed in aq. NaOH solution, neutralised with acid and reacted with the corresponding LnCl_3_ salt. The desired **[LnL^1^]** complexes were purified by reverse-phase HPLC.

### Human α_1_-AGP binding studies of **[EuL^1^]** and **[TbL^1^]**

The complexes **[TbL^1^]** and **[DyL^1^]** each showed a characteristic ion-centred emission signature, whilst **[EuL^1^]** showed very low emission intensity. As significant residual ligand fluorescence was not observed, the quenching of emission in **[EuL^1^]** is likely to be due to competitive depopulation of the ligand singlet excited state by formation of a ligand-to-metal charge transfer state. Bubbling argon gas through the aqueous solution of **[EuL^1^]** altered neither the magnitude nor the lifetime of the observed emission, consistent with the singlet nature of the hypothesised ICT state. By decreasing the polarity of the solvent, the total emission intensity was significantly enhanced, with some changes in the Eu emission spectral signature observed in less polar solvents, such as THF and 1,4-dioxane (Fig. S1†). Emission intensity variations did not correlate with changes in solvent viscosity. The measured lifetime of the excited state also did not vary, negating the involvement of a twisted intramolecular charge transfer (TICT) state in the emission quenching process.

A similar solvent-dependent behaviour was observed in recent work, where the ICT state in the related europium complex, **[EuL^3^]**^13^ (Fig. 2), demonstrated a strong solvent dependence of emission. Indeed, a near exponential increase of the total emission intensity was found as a function of Reichardt's solvent polarity parameter.^13^ Furthermore, it was revealed that addition of HSA resulted in a 100-fold amplification of the signal intensity, attributed to the significant polarity change in the drug-binding pocket.

**Fig. 3:**
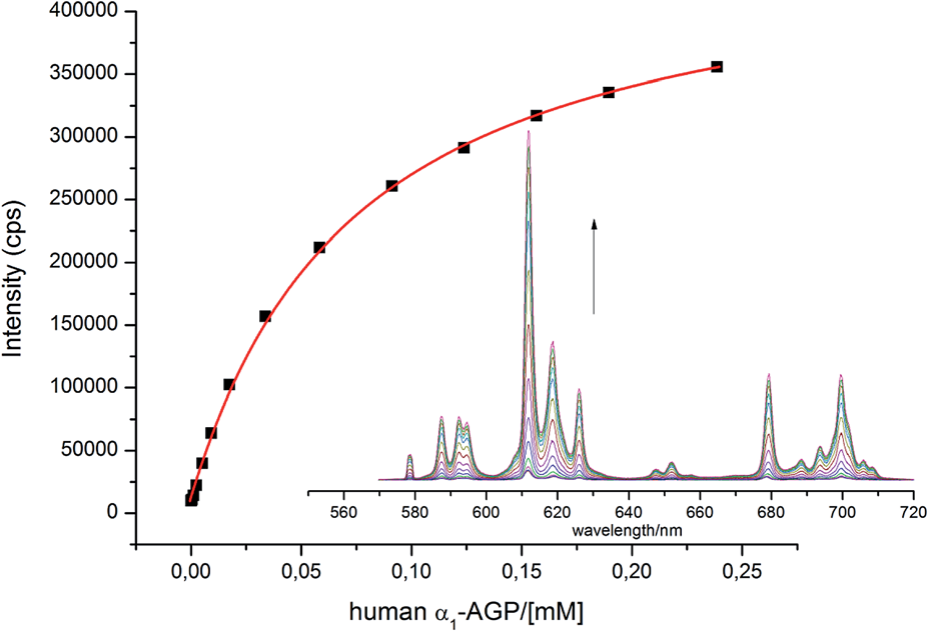
Change of the total emission intensity upon addition of human α_1_-AGP to **[EuL^1^]**; (**[EuL^1^]** 9 μM; log *K* = 4.1(01), assuming a 1 : 1 binding isotherm, *λ*_ex_ = 310 nm, 298 K, pH = 7.40).

With this behaviour in mind, similar protein binding studies were carried out for **[EuL^1^]**, examining human and bovine serum albumins, γ-ImG, fibrinogen, α_1_-AGP (bovine and human forms) and α_1_-AAT. Only addition of α_1_-AGP (human and bovine) revealed a substantial increase in the total europium emission intensity, with apparent binding constants log *K* = 4.1 and 4.7 estimated for the human and bovine species respectively (Fig. 3). Only for the bovine species was a significant change in the spectral signature observed (0.1 M HEPES, pH = 7.40).

With added human α_1_-AGP, **[EuL^1^]** showed an induced CPL signal that was almost identical to that observed for **[EuL^3^]** with added HSA. A closer inspection of the structure of **[EuL^1^]** reveals a coordination environment almost identical to **[EuL^3^]**, with two carboxylate oxygen atoms, a sulphonamide nitrogen atom and a pyridine nitrogen atom bound to the Eu^3+^ ion. As less significant change of the spectral signature was observed upon addition of human α_1_-AGP, perturbation of the primary coordination environment is unlikely to have occurred and the observed induced CPL can be attributed to the preferential stabilisation of one of the two complex enantiomers, Λ(λλλλ) and Δ(δδδδ), which exist in equilibrium in aqueous solution. To assign the observed CPL signature to one of these enantiomers, a comparison of the CPL spectrum with previously reported spectra of chiral europium complexes was carried out.^14^ Such an analysis resulted in a tentative Δ (δδδδ) assignment, (Fig. S9†).

**Scheme 1:**
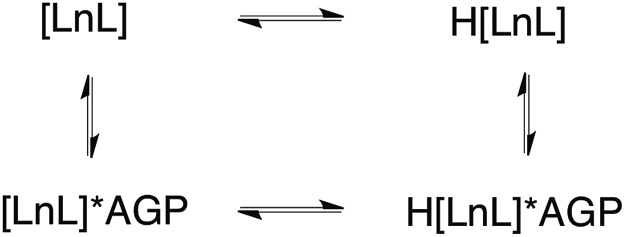
Equilibria present in the **[LnL^1^]**/α_1_-AGP system, showing the protonated and non-protonated forms.

The sulphonamide arm binding behaviour determines the pH changes in the emission spectrum.^15^–^18^ Reversible binding of the nitrogen to Eu^3+^ ion occurs in solution with protonation leading to addition of coordinated water molecules (Scheme 1, and *vide infra*). Addition of certain anions and proteins can significantly alter the position of the equilibrium, affecting the p*K*_a_ associated with protonation of the sulphonamide nitrogen, as a result of competitive binding.^15^ As only a very low emission intensity was observed for **[EuL^1^]** without any added α_1_-AGP, the terbium analogue **[TbL^1^]** was also used to investigate the protonation behaviour of the complex.

Only minor changes to the terbium emission pattern were observed upon lowering the pH from 8.0 to 3.0, whilst the total emission intensity decreased, consistent with a replacement of the sulphonamide nitrogen atom by two water molecules. At the same time, the lifetime of the terbium ^5^D_4_ excited state decreased from 1.85 ms to 1.14 ms, in line with the more efficient non-radiative quenching *via* energy transfer to the proximate OH oscillators associated with the coordinated waters. The apparent p*K*_a_ of the free Tb complex (4.2(±0.1)) was similar to that measured for **[EuL^1^]** when bound to human α_1_-AGP (4.6(±0.1)).

Protein addition for **[TbL^1^]** and **[EuL^1^]** increased the lifetime of the excited state, consistent with the absence of water molecules bound to the lanthanide ion at both lower and ambient pH (*q* = 0). At lower pH, *q* is also zero; such behaviour suggests that the bound water was replaced with a carboxylate oxygen (*e.g.* the side chain carboxylate oxygen of Glu-64/65 for human and potentially bovine AGP, respectively) in the protein bound form of the complex (Table 1).

**Table 1:** Lifetimes of the ^5^D_0_ excited state (in H_2_O and D_2_O) and hydration numbers (*q*) for **[EuL^1^]** in the presence of human and bovine α_1_-AGP, as well as for **[TbL^1^]** with no added protein

	H_2_O/ms	D_2_O/ms	*q*
Human α_1_-AGP	0.78 (pH 7.0)	1.04 (pD 7.4)	0.1
	0.91 (pH 3.0)	1.17 (pD 3.4)	0
Bovine α_1_-AGP	0.22 (pH 9.8)	0.22 (pD 10.4)	0^*b*^
	0.82 (pH 6.0)	1.33 (pD 6.4)	0.3
No protein^*a*^	1.80 (pH 7.0)	1.98 (pD 7.4)	0
	1.14 (pH 3.0)	1.92 (pD 3.4)	1.5

^*a*^Obtained for **[TbL^1^]**; Tb emission lifetimes with added human AGP were 2.06 (pH 4.1) and 1.63 ms (pH 8.1), and 1.78 ms with added bovine AGP (pH 6.5), consistent with *q* = 0 in each case.

^*b*^At high pH/pD quenching by charge transfer inhibits the use of the normal method of lifetime analysis to assess *q* values, for which the dominant quenching mechanism must only involve vibrational energy transfer to OH oscillators.

**Fig. 4:**
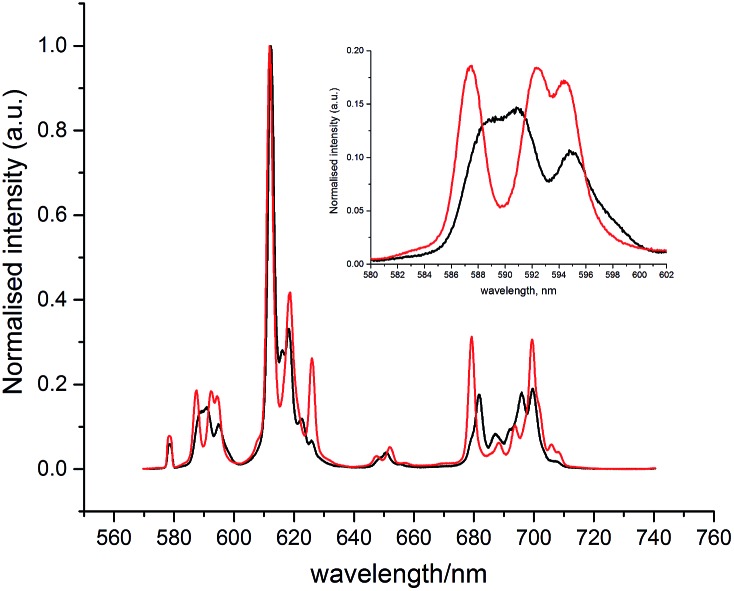
Changes of the emission spectrum of **[EuL^1^]** in the presence of human α_1_-AGP at pH = 3.0 (SAP, black) and pH = 6.0 (TSAP, red) (**[EuL^1^]** 8 μM, α_1_-AGP 25 μM, *λ*_ex_ = 310 nm, 298 K).

With the human α_1_-AGP bound **[EuL^1^]** complex, a concomitant change of the spectral signature with pH was observed. The most remarkable change occurred in the ^5^D_0_ → ^7^F_1_ transition (Fig. 4), where a change in the sign of the crystal field parameter *B*20 was observed, by analysing the nature of the Δ*J* = 1 manifold.^19^,^20^ The values of *B*20 and *B*22 (*i.e.* defined in terms of spherical tensor analysis),21*b* were –556 and –117 cm^–1^ for the high pH form, and +448 and +92 cm^–1^ in the protein bound state.

These findings can be explained in terms of the transition between the TSAP (with the sulphonamide nitrogen bound at higher pH) and SAP (with a dissociated sulphonamide nitrogen) coordination geometries. Attempts to trace these changes by following chemical shifts in the ^1^H NMR spectrum as a function of pH were not successful, as very broad signals were observed at lower pH values, probably as a result of the fast exchange between conformational isomers in the SAP configuration.

### NMR analysis of complex structure

**Fig. 5:**
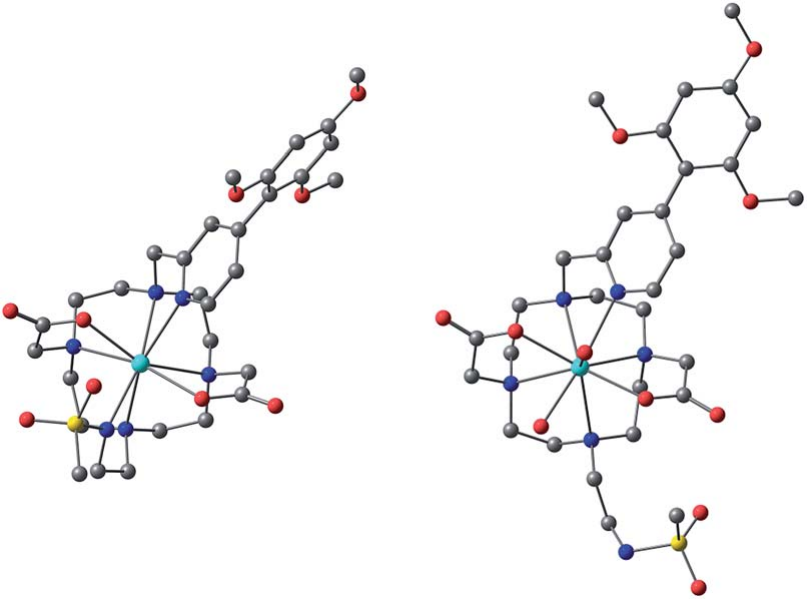
Optimised structures of **[YL^1^]** in octadentate twisted square antiprismatic (TSAP) coordination geometry and **[HYL^1^(H_2_O)_2_]^+^** in 9-coordinate mono-capped square-antiprismatic coordination (SAP).

The simulation of NMR pseudocontact shift data (PCS) is a valuable tool to evaluate the quality of the proposed molecular structure of a lanthanide complex in solution, by comparing calculated values with experimentally observed shifts. It has been previously shown that predicted PCS values based on an optimised X-ray structure of the complex were in a good agreement with experimentally observed ones.^21^ As no crystal structure was available for **[LnL^1^]**, DFT calculations were employed instead, and the final structures are shown above (Fig. 5). Lifetime measurements had shown that reversible protonation of the sulphonamide nitrogen at lower pH replaced it with two water molecules directly bound to the lanthanide ion, increasing its coordination number from 8 to 9. The concomitant changes of the sign of *B*20 term, as evident from the emission spectrum of Eu (Fig. 4), suggested the change of coordination environment between TSAP and SAP configurations. To verify this assumption, DFT simulations of structures of the 8-coordinate TSAP and 9-coordinate SAP isomers (Fig. 5), augmented by pseudo-contact shift fitting of **[EuL^1^]** and **[DyL^1^]** were carried out. In addition, ^1^H NMR and ^1^H–^1^H COSY spectra for the TSAP isomer were measured, (Fig. S2 and S5†).

**Fig. 6:**
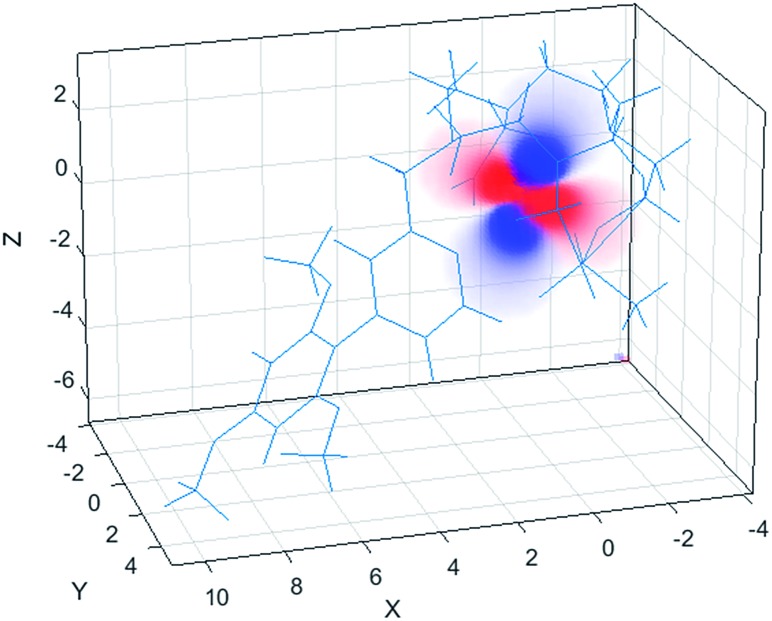
Pseudocontact shift fields for the **[DyL^1^]** complex, reconstructed by Spinach using the best-fit magnetic susceptibility tensor. Positive PCS is shown in red, negative – in blue, transparency indicates the absolute value.

**Fig. 7:**
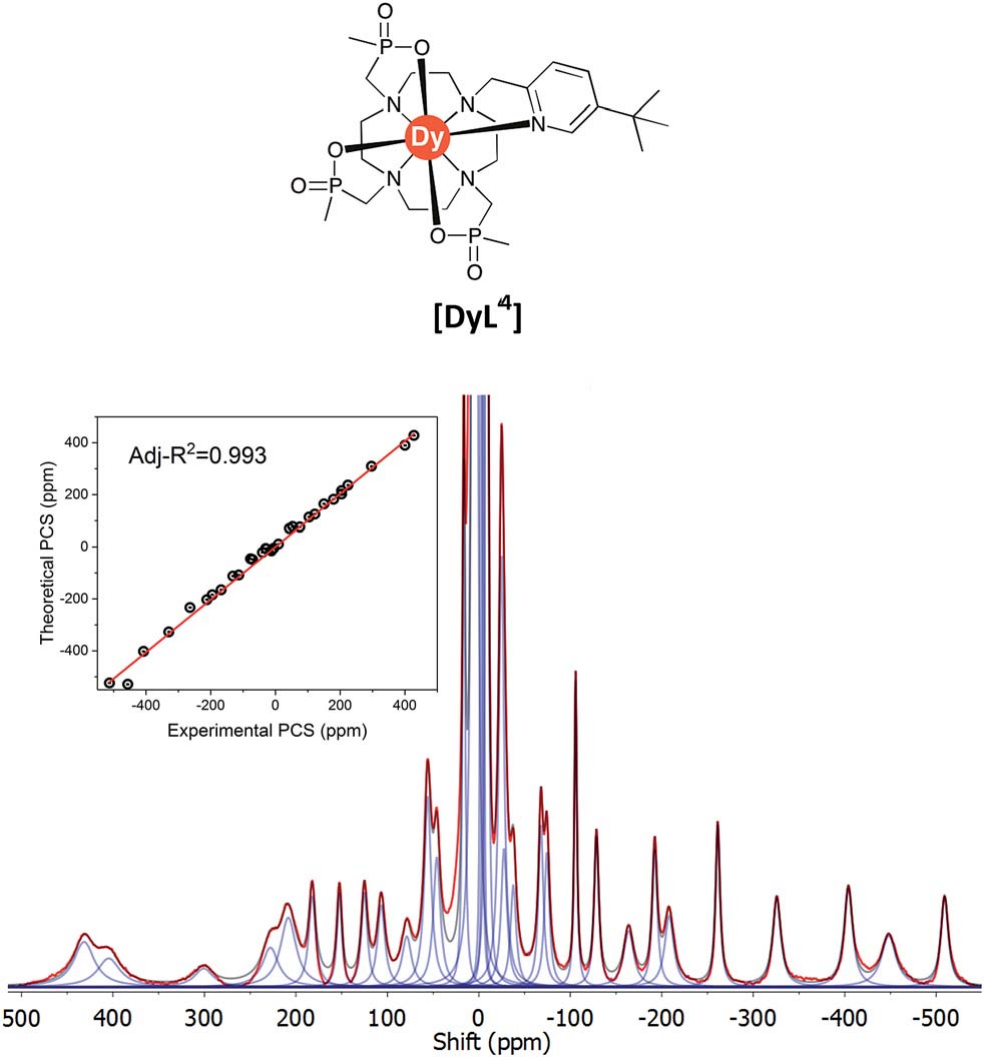
Experimental ^1^H NMR spectrum of **[DyL^1^]** (magenta) and a simulated spectrum (blue); (1T, 300 K, D_2_O) showing the comparison of theoretical and experimental pseudocontact shift values (PCS) (Tables S1 and S3†).

The ^1^H NMR spectrum (Fig. S2†), ^1^H–^1^H NOESY (Fig. S3†) and ^1^H–^1^H ROESY (Fig. S4†) spectra of **[YL^1^]** were used to estimate the diamagnetic contribution to the chemical shifts. A simulated structure of the TSAP isomer for **[DyL^1^]** in particular produced a very good fit (Fig. 6 and 7, Tables 2 and S1–S4†). Since exchange broadened signals were obtained at lower pH values for the SAP structure, no fitting of its simulated structure was possible as no PCS values were recorded for the SAP isomer.

**Table 2:** Magnetic susceptibility tensors (SI units) for the complex **[DyL^1^]** in comparison to the related TSAP triphosphinate-mono-pyridyl complex **[DyL^4^]**,^21^ obtained by paramagnetic shift fitting and computed by CASSCF (in parentheses), expressed in terms of axiality (*χ*_ax_), rhombicity (*χ*_rh_) and Euler angles

Complex	*χ*_ax_/Å^3^	*χ*_rh_/*χ*_ax_	*α*/°	*β*/°	*γ*/°
**[DyL^1^]**	–0.55	0.23	185	20	207
**[DyL^4^]**	–0.57, (–0.59)	0.30, (0.25)	189, (198)	20, (24)	201, (14)

The best-fit magnetic susceptibility tensor (Table 2) and the resulting PCS field (Fig. 6) are very similar in size and orientation to those found with **[DyL^4^]**,21a,b consistent with their very similar TSAP coordination geometries (Fig. 5).

In Bleaney's theory of magnetic anisotropy,21c,d ligand field parameters can be computed from the axiality and rhombicity of the magnetic susceptibility, at the high temperature limit.1
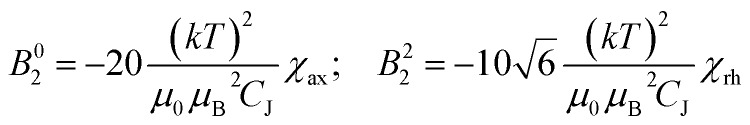



According to eqn (1), the ligand field parameters *B*20 and *B*22 are estimated to be –486 and –137 cm^–1^, which is in quite good agreement with the parameters extracted from the europium emission spectra. The small variation might be due to the differences in the radial wavefunction between Eu(iii) and Dy(iii) or because of the high temperature limit approximation.

The lifetime data had shown that there were no bound water molecules in the presence of added protein (Table 1). However, the positive sign of the *B*20 term was evident analysing the europium emission spectrum of the protein bound form, consistent with a very different coordination type that is adopted when chelating, for example, the carboxylate oxygens of a proximate Glu or Asp side chain.

Tables of experimental and calculated shifts are given in the ESI,† together with optimized structural coordinates for the Y, Eu, Tb and Dy complexes of L^1^ in the TSAP form and the 9-coordinate SAP structure with the unbound sulphonamide N atom and two added waters for Y and Eu. The Y, Eu, Tb and Dy complexes of L^1^ are isostructural.

### Bovine α_1_-AGP binding studies of **[EuL^1^]** and **[TbL^1^]**

**Fig. 8:**
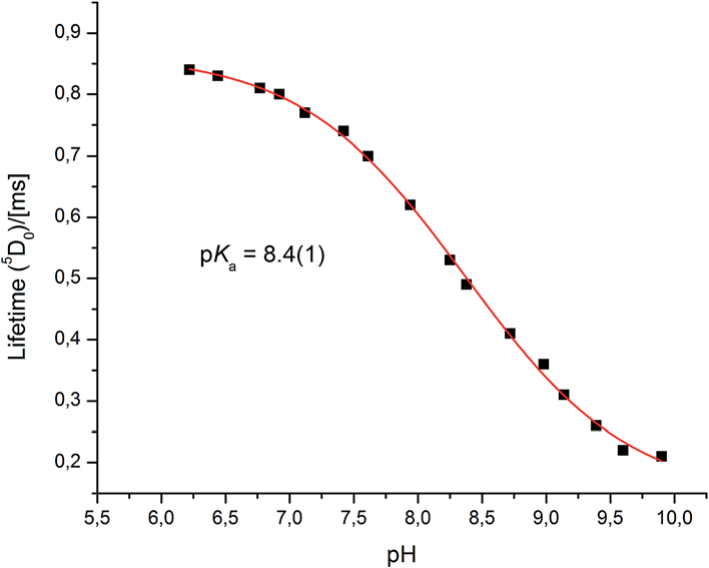
The pH dependence of europium emission in bovine α_1_-AGP bound complex **[EuL^1^]** (295 K, 0.1 M NaCl, 5 μM **[EuL^1^]**, 25 μM α_1_-AGP).

Very different emission behaviour was observed in the presence of bovine α_1_-AGP, where a change of the spectral signature was observed upon binding the protein to **[EuL^1^]** (0.1 M HEPES, pH = 7.40). At this pH, the resulting emission spectral form resembled that observed for **[EuL^1^]** with added human α_1_-AGP, when the sulphonamide nitrogen was dissociated and replaced with carboxylate oxygen atoms of the side chain amino-acid. This behaviour initially suggested a higher p*K*_a_ value for **[EuL^1^]** with added bovine α_1_-AGP (Fig. S6–S8†). Indeed, an apparent p*K*_a_ of 8.4(±0.1) was calculated (Fig. 8). This value is much higher than that observed for **[TbL^1^]** with no added protein (4.2(±0.1)), (Fig. S13†).

Both the total emission intensity and the lifetime of the excited state experienced a significant reduction, with the latter decreasing from *τ* = 0.84 ms (pH 6.2), to *τ* = 0.22 ms (pH 9.9). The observed strong quenching of emission at higher pH, along with the elevated p*K*_a_ value with added bovine (but not human) α_1_-AGP suggests a different interaction between **[EuL^1^]** and the amino-acids in the bovine drug-binding pocket.

**Fig. 9:**
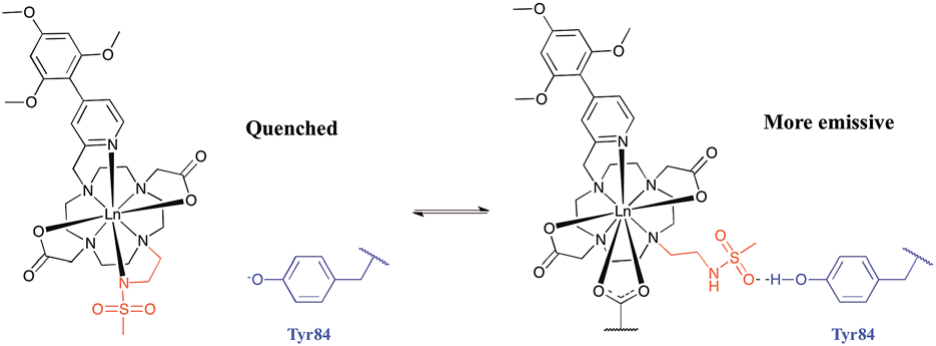
Rationalisation of the enhanced p*K*_a_ observed for **[LnL^1^]** when bound to bovine α_1_-AGP. In the sulphonamide bound complex, the proximate phenolate quenches the luminescence by electron transfer.

The absence of a crystal structure of bovine α_1_-AGP makes it difficult to be sure about which amino-acids are close in space to the drug-binding pocket. However, a simulated computer model of the bovine α_1_-AGP tertiary structure22*a* suggests a significant correspondence in the structures of human and bovine variants of the protein, (Fig. S14†), and therefore amino-acid residues that may be close to the bound drug are expected to have rather similar positions in the 3D structure. On examining those residues that are close to the expected drug-binding site, the aromatic amino-acid Tyr-84 was pinpointed in bovine α_1_-AGP; in the human form the nearest Tyr residue is Tyr-78. The side chain phenol can potentially bind to a sulphonamide oxygen, through formation of a stabilising hydrogen bond. When this phenol is deprotonated, *i.e.* at higher pH, the sulphonamide arm can no longer interact with Tyr-84, as the sulphonamide nitrogen atom is coordinated to Eu^3+^ (Fig. 9). At the same time, the electron rich phenolate of Tyr-84 can quench **[EuL^1^]** emission by a charge transfer mechanism that also occurred, but to a lesser degree with **[TbL^1^]**. It is therefore plausible that it is the protonation of Tyr-84 that is being observed in the pH dependence of Eu emission (Fig. 8). The lowered phenol p*K*_a_ from its normal value around 9.7, can be attributed to either an electrostatic field effect, causing stabilisation of the conjugate base by proximate cationic side chains (*e.g.* Lys-82, Arg-85), or by a stabilising H-bonding interaction with a proximate H-bond donor.22*b* In the human form of AGP, the nearest Tyr (Tyr-78) in contrast is flanked by threonine, glutamine and leucine residues so no such field effect seems possible. Previous examples of the quenching of sensitised Eu emission by electron rich species, have included urate, ascorbate and various catecholates.^23^

**Fig. 10:**
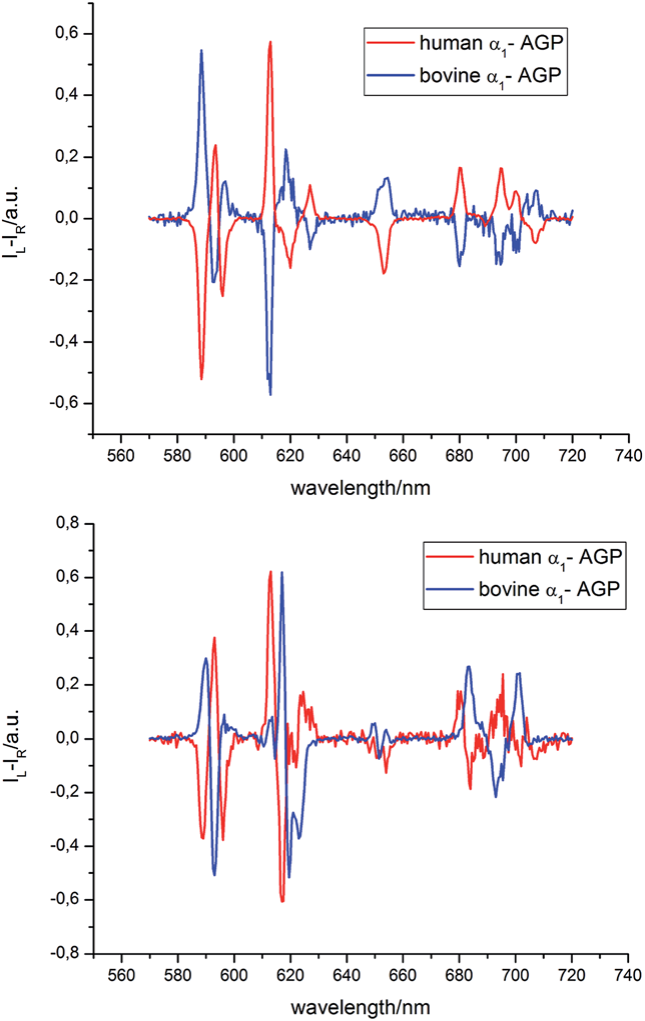
Induced CPL spectra of **[EuL^1^]** bound with human and bovine α_1_-AGP, when the sulfonamide arm is coordinated (pH = 9.3, top) and when it is not bound (pH = 3.6, lower) to the Eu^3+^ ion (*λ*_ex_ = 310 nm, D_2_O, 295 K).

The analysis of the induced CPL spectrum of **[EuL^1^]** with added bovine α_1_-AGP at higher pH revealed a spectrum of opposite sign to that observed for **[EuL^1^]** with added human α_1_-AGP, under the same conditions (Fig. 10 and S9†). This surprising observation suggests the stabilisation of the complex with opposite helicity (*i.e.* the Λ(λλλλ) enantiomer) for **[EuL^1^]** when it is located within the drug-binding pocket of bovine α_1_-AGP. Upon lowering the pH of the solution, the CPL spectrum also changed, and resembled the inverted signature of **[EuL^1^]** with added human α_1_-AGP, although not completely matching it. These small differences in the CPL and total emission signatures may be caused by differences between the amino-acid moieties, (*e.g.* a Glu or Asp side chain O) which bind to **[EuL^1^]** at lower pH values, when the sulphonamide arm is dissociated.

Similar behaviour was observed for **[TbL^1^]** with added human and bovine α_1_-AGP (Fig. S10†), when opposite, but not identical CPL spectral signatures were found for the unbound sulphonamide. At elevated pH, when the sulphonamide N atom is coordinated, very weak induced CPL signals were observed for each protein. Because **[TbL^1^]** was emissive in the absence of protein, and since addition of bovine α_1_-AGP induced a strong CPL signal, the change in the dissymmetry ratio, *g*_em_, could be monitored as a function of added protein concentration (Fig. S11†). Analysis of the resultant binding isotherm revealed an affinity constant log *K* = 5.1, which is a somewhat higher value than that calculated by following the total emission intensity variation of **[EuL^1^]** (log *K* = 4.7).

### Ratiometric monitoring of α_1_-AGP levels in serum

**Fig. 11:**
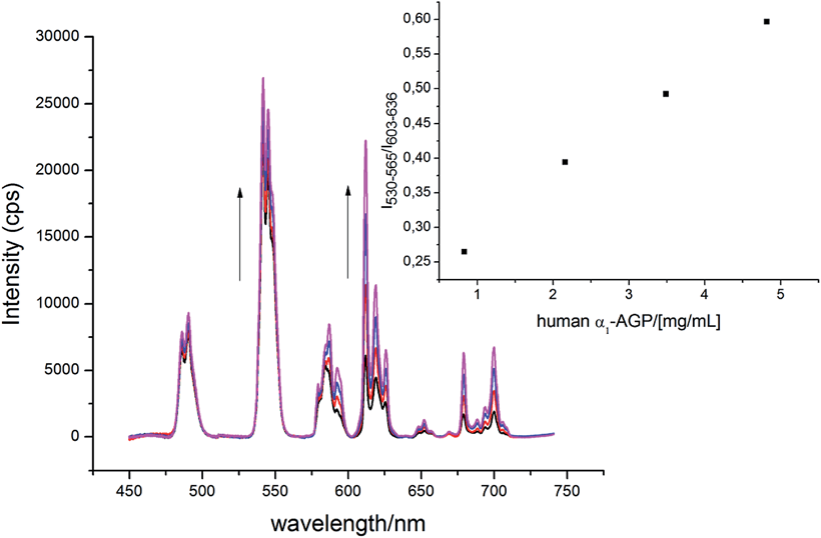
Change of the total emission spectrum of **[EuL^1^]**/**[TbL^1^]** (22 μM/2 μM) ‘cocktail’ upon addition of human α_1_-AGP to human serum (pH = 7.40, *λ*_ex_ = 310 nm, 298 K).

The α_1_-AGP concentration in plasma can experience up to a four-fold increase from normal values, in response to inflammatory stimulus. Accordingly, to foetal calf or human serum samples was added a mixture of **[EuL^1^]** and **[TbL^1^]** (22 μM/2 μM) and the variation of the emission intensity ratio was monitored upon incremental addition of bovine and human α_1_-AGP, respectively (Fig. 11 and S21†). In each case, a quasi-linear dependence between the relative intensities of the Tb ^5^D_4_ → ^7^F_5_ band in **[TbL^1^]** and the ^5^D_0_ → ^7^F_2_ transition in **[EuL^1^]** with the concentration of α_1_-AGP was observed. A lower apparent binding affinity was noted, compared to those measured in salt and buffer solution only. This discrepancy might be explained by competitive binding of **[EuL^1^]** and **[TbL^1^]** to serum albumin, decreasing the effective concentration of α_1_-AGP.

Even though preliminary studies revealed no ‘switch-on’ response for **[EuL^1^]** upon addition of human and bovine serum albumin, a more detailed analysis of its effect on the total emission intensity of **[TbL^1^]** was carried out. In each case, (HSA and BSA), quenching of the total emission intensity was observed when excess of the protein was added, with no visible change of the spectral signature detected. Similar binding constants were observed with added HSA and BSA (log *K* = 3.4 and log *K* = 3.2, respectively, Fig. S22 and S23†), which are two orders of magnitude lower than the corresponding binding constants for this complex with α_1_-AGP. Notwithstanding this difference in binding constants, the occurrence of a significantly higher concentration of serum albumin (*ca.* 0.6 mM) than α_1_-AGP (0.02 mM) most likely results in a noticeable quenching of both the Tb and Eu excited states by charge transfer from serum albumin.

**Fig. 12:**
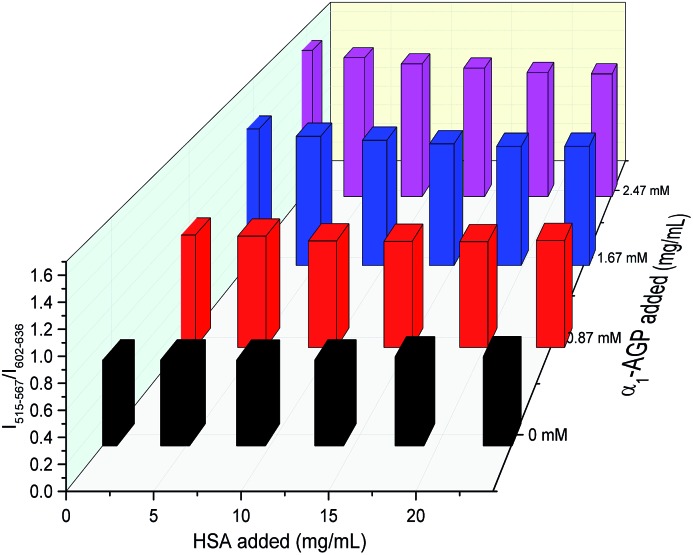
Variation of the relative intensity of emission bands at 515–567 nm and 602–636 nm of the mixture of **[EuL^1^]** and **[TbL^1^]** (21 μM/0.8 μM) as a function of added HSA and human α_1_-AGP (*λ*_ex_ = 310 nm, 298 K).

To account for serum albumin quenching, a three-dimensional titration chart was plotted by considering simultaneous variation of the concentration of both human serum albumin and human α_1_-AGP. The resulting plot, (Fig. 12), revealed the rising trend for the ^5^D_4_ → ^7^F_5_/^5^D_0_ → ^7^F_2_ intensity ratio at any given concentration of HSA, although the slope of the plotted curve is in turn a function of the HSA concentration. The dependence is shallower at higher concentrations of HSA, consistent with more efficient competitive binding of **[EuL^1^]** and **[TbL^1^]** to HSA as its concentration rises. The described system could be improved further by introducing a third component, which has a different spectral response to both HSA and human α_1_-AGP compared to **[EuL^1^]** and **[TbL^1^]**, or one that is non-responsive to the presence of each of the two proteins. Such a system could then provide simultaneous monitoring of the concentration of both proteins in serum, allowing a calibration surface for variable concentrations of HSA and human α_1_-AGP to be defined.

### Competitive drug-binding studies

In contrast to serum albumin, only one well-defined binding pocket is present in α_1_-AGP. This pocket can permit the transport of essential drugs in plasma, and in several cases the drug is preferentially bound to serum albumin. It has been shown recently that a luminescent europium complex can be efficiently used for determining binding constants of selected drugs to human α_1_-AGP, by competitive replacement of the luminescent complex bound to the protein with added drug, accompanied by the changes in the total emission intensity and induced CPL signal.^12^ The determined binding constants were in good agreement with previously reported values, corroborating the applicability of this approach.

**Fig. 13:**
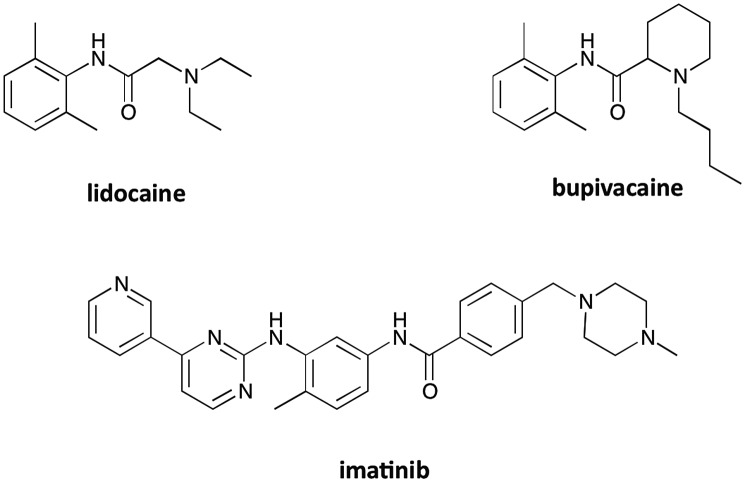
Molecular structures of drugs used in the present study.

In the present study, three common drugs – imatinib, lidocaine and bupivacaine (Fig. 13) – were used to examine competitive binding between **[EuL^1^]** and bovine or human α_1_-AGP (Table 3). Addition of lidocaine (Fig. S15†) and bupivacaine (Fig. S17†) to human α_1_-AGP with bound **[EuL^1^]** revealed binding constants (log *K* = 4.4 and 5.6 respectively) similar to those previously reported (log *K* = 4.4 and 5.7 respectively). A very similar value was observed in analogous experiments with bovine α_1_-AGP (log *K* = 4.5) for lidocaine (Fig. S16†), although the value for bupivacaine (Fig. S18†) was found to be significantly higher (log *K* = 6.4). In each case, the total emission intensity of **[EuL^1^]** returned to the initial value observed in the absence of added protein, when an excess of the drug has been added.

**Table 3:** Binding affinities of **[EuL^1^]**, lidocaine, bupivacaine and imatinib to bovine and human α_1_-AGP (pH = 7.40, 298 K). Literature values are given in parentheses;^24^,^25^ no literature data is available for the bovine AGP drug affinities

	**[EuL^1^]**	Lidocaine	Bupivacaine	Imatinib
Human α_1_-AGP	4.1	4.5 (4.4)	5.6 (5.7)	5.6 (6.4)
Bovine α_1_-AGP	4.7	4.5	6.4	4.8

A different situation was observed when imatinib (Fig. S19 and S20†) was added to the proteins with bound **[EuL^1^]**. Addition of imatinib did not seem to completely displace bound **[EuL^1^]**, as the spectral signature of the europium complex and the dissymmetry factor of the terbium analogue **[TbL^1^]** bound to bovine α_1_-AGP did not change upon addition of the drug. Emission quenching was observed for both europium and terbium complexes, and the total emission intensity of **[EuL^1^]** with excess of added drug was lower than for the complex without added protein, suggesting an efficient non-radiative quenching of the biaryl chromophore by imatinib inside the drug-binding pocket (*e.g.* by a π–π stacking interaction).

Significantly lower binding constants were calculated for the binding of imatinib to human α_1_-AGP (log *K* = 5.6 *vs.* log *K* = 6.4), and this was even an order of magnitude lower (log *K* = 4.8) in the case of bovine α_1_-AGP.

The relatively low binding constant of **[EuL^1^]** to human and bovine α_1_-AGP permitted competitive binding experiments to be carried out, *e.g.* with lidocaine and bupivacaine – drugs that both posses higher binding affinities towards α_1_-AGP than **[EuL^1^]**. In spite of the differences between human and bovine α_1_-AGP in their amino-acid content near the drug-binding site, similar binding constants were found in each case. The terbium analogue, **[TbL^1^]**, with a strong induced CPL signal when bound to bovine α_1_-AGP can itself be used for monitoring drug-binding, by following the variation of the dissymmetry factor.

## Conclusions

Water-soluble and pH-responsive macrocyclic lanthanide complexes with a biaryl chromophore exhibit a high affinity towards the human and bovine forms of the serum protein, α_1_-AGP. The emission of **[EuL^1^]** is quenched in aqueous solution in the absence of the protein, and showed a 30-fold increase in emission intensity upon adding α_1_-AGP. Furthermore, this pH-responsive probe showed a strong CPL signal, which intriguingly showed opposite signs for the human and bovine variants of α_1_-AGP. As the **[EuL^1^]** and **[TbL^1^]** complexes showed different sensitivity towards added α_1_-AGP, their mixture could be used in creating a ratiometric system to determine the concentration of α_1_-AGP, in either human or bovine serum. Competitive binding studies with popular prescription drugs showed little difference in affinity between human and bovine variants of α_1_-AGP.

Changes in the emission spectral signature of the europium complex as a function of pH revealed the reversible switch between a twisted square antiprismatic structure (TSAP) at higher pH and a mono-capped square antiprismatic (SAP) coordination at lower pH. The corresponding structures of the Eu and Dy systems were simulated using a combined DFT and NMR pseudocontact shift (PCS) fitting analysis, estimating the pseudocontact shifts from the behaviour of the diamagnetic analogue **[YL^1^]**. The simulation allowed the full magnetic susceptibility tensor for **[DyL^1^]** to be calculated, and it was found to be remarkably similar to that recently found for the complex **[DyL^4^]** which also possesses an eight-coordinate TSAP structure. Taken together, such NMR and computational analyses are in agreement with the large change in solution structure revealed by emission analysis that allows the assessment of the europium ligand field parameters, *B*20 and *B*22, whose sign changes upon the TSAP and SAP interconversion. Indeed, in the past, such behaviour has either been overlooked or misapprehended,^26^ and yet it can explain the very dramatic changes in paramagnetic NMR and lanthanide emission spectral behaviour that can characterise both large21*b* and surprisingly, even more subtle ligand field perturbations.^27^

## Conflicts of interest

There are no conflicts to declare.

## Supplementary Material

Supplementary information
