# Supplementary material for: Chiral probes for α1-AGP reporting by species-specific induced circularly polarised luminescence
Source: Chem Sci. 2018 Feb 19;9(11):2996–3003. doi: 10.1039/c8sc00482j (PMC5915836; doi:10.1039/c8sc00482j)
Supplement: Supplementary file 1 [file SC-009-C8SC00482J-s001.pdf]

Electronic Supplementary Material (ESI) for Chemical Science.
This journal is © The Royal Society of Chemistry 2018

Supporting Information

Chiral probes for α_1-AGP reporting by species-specific induced circularly polarised luminescence

Sergey Shuvaev, Elizaveta A. Suturina, Kevin Mason, and David Parker

Table of Contents

Experimental Procedures **2**
Synthesis **4**
Scheme 1. Synthetic procedure for **[LnL^1]** 4
Figures and tables **9**
Fig. S1 Emission spectra of **[EuL^1]** in different organic solvents and water 9
Fig. S2 ^{1}H NMR spectra of **[EuL^1]** and **[YL^1]** 9
Fig. S3 ^{1}H-^{1}H NOESY spectrum of **[YL^1]** 10
Fig. S4 ^{1}H-^{1}H ROESY spectrum of **[YL^1]** 10
Fig. S5 ^{1}H-^{1}H COSY spectrum of **[EuL^1]** 11
Fig. S6 Change of the total emission intensity upon addition of bovine α_1-AGP to **[EuL^1]** 11
Fig. S7 Change of the total emission intensity upon addition of **[EuL^1]** to bovine α_1-AGP 12
Fig. S8 Change of the total emission intensity upon addition of **[EuL^1]** to human α_1-AGP 12
Fig. S9 Comparison between CPL spectra recorded for **[EuL^1]** in the presence of human and bovine α_1-AGP at pH = 9.30 and CPL spectra of complexes reported in [10] 13
Fig. S10 Induced CPL spectra of **[TbL^1]** bound with human and bovine α_1-AGP, when the sulfonamide arm is unbound 13
Fig. S11 Change of the emission dissymmetry factor upon addition of bovine α_1-AGP to **[TbL^1]** .. 14
Fig. S12 pH calibration curves for **[EuL^1]** with human α_1-AGP 14
Fig. S13 pH calibration curves for **[TbL^1]** 15
Fig. S14 Alignment of the sequences of various forms of α_1-AGP (F. Ceciliaini et al, *Vet. Res.* 2005, **36**, 735-746) 15
Fig. S15 Variation of europium emission intensity in the presence of human α_1-AGP showing the fit to the data points with added lidocaine 16
Fig. S16 Variation of europium emission intensity in the presence of bovine α_1-AGP showing the fit to the data points with added lidocaine 16
Fig. S17 Variation of europium emission intensity in the presence of human α_1-AGP showing the fit to the data points with added bupivacaine 17
Fig. S18 Variation of europium emission intensity in the presence of bovine α_1-AGP showing the fit to the data points with added bupivacaine 17
Fig. S19 Variation of europium emission intensity in the presence of human α_1-AGP showing the fit to the data points with added imatinib 18
Fig. S20 Variation of europium emission intensity in the presence of bovine α_1-AGP showing the fit to the data points with added imatinib 18
Fig. S21 Change of the total emission spectrum of **[EuL^1]**/**[TbL^1]** 'cocktail' upon addition of human α_1-AGP to human serum 19
Fig. S22 Quenching of the total emission intensity of **[TbL^1]** upon addition of HSA. 19
Fig. S23 Quenching of the total emission intensity of **[TbL^1]** upon addition of BSA 20
Fig. S24 Variation of the total emission intensity of **[DyL^1]** as a function of added human α_1-AGP20
Table 1 Summary of the ^{1}H NMR solution data (D_2O, 300K, 43 MHz) and the best fitted values . 21

Table 2 Optimized structures of **[LnL^1]** in the eight coordinate twisted square antiprismatic coordination geometry (TSAP), that defines the structure of the sulphonamide bound form that is observed in solution at higher pH values. 21

Table 3 Best fit parameters of the traceless susceptibility tensor (Å^3 SI units) for [DyL^1] obtained from the fit of the experimental PCS data measured at 1 Tesla and 27 °C.

Table 4 Optimised structures of **[HLnL^1(H$_2$O)$_2$]$^+$**: a 9-coordinate mono-capped square antiprismatic geometry (SAP). 23

References **24**

Experimental Procedures

Reagents and solvents were purchased from commercial suppliers (Sigma-Aldrich, Fluorochem, Alfa Aesar, Fisher Scientific, Apollo Scientific) and used as delivered without further purification. Air and/or moisture sensitive reactions were carried out in argon atmosphere using anhydrous solvents. ^{1}H and ^{13}C NMR spectra were recorded on Bruker Avance 400 (^{1}H at 400.06 MHz, ^{13}C at 100.61 MHz), Varian VNMRS-600 (^{1}H at 599.67 MHz, ^{13}C at 150.79 MHz) and Varian VNMRS-700 (^{1}H at 699.73 MHz, ^{13}C at 175.95 MHz) spectrometers. ^{1}H and ^{13}C NMR chemical shifts are referenced to the signals of the solvent ($CDCl_3$). Electrospray mass spectra were obtained on a TQD mass spectrometer equipped with an Acquity UPLC system, an electrospray ion source, and an Acquity photodiode array detector (Waters Ltd., U.K.). Accurate masses were recorded on an LCT Premier XE mass spectrometer or a QToF Premier Mass spectrometer, both equipped with an Acquity UPLC system, a lock-mass electrospray ion source, and an Acquity photodiode array detector (Waters Ltd., U.K.). Methanol or acetonitrile was used as the carrier solvent. Reverse-phase preparative HPLC was performed at 295 K using a Shimadzu system consisting of a Degassing Unit (DGU-20A5R), a Prominence Preparative Liquid Chromatograph (LC-20AP), a Prominence UV/Vis Detector (SPD-20A) and a Communications Bus Module (CBM-20A). An XBridge C18 OBD 19 x 100 mm, i.d. 5 mM column was used with a flow rate of 2 mL min^{-1} (analytical) or 17 mL min^{-1} (prep). The solvent system was H_2O + 0.1% formic acid/MeOH (MeCN) + 0.1% formic acid (gradient elution). All optical analyses were carried out in quartz cuvettes with a path length of 1 cm. UV/Vis absorbance spectra were measured on an ATI Unicam UV/Vis spectrometer (Model UV2) using Vision software (version 3.33). Emission spectra were recorded using an ISA Jobin-Yvon Spex Fluorolog-3 luminescence spectrometer with a CCD detector using FluorEssence software. Lifetime measurements were carried out using a Perkin Elmer LS55 spectrometer using FL Winlab software. Quantum yields were calculated by comparison with known standards. CPL spectra were recorded on a custom built spectrometer consisting of a laser driven light source (Energetiq EQ-99 LDLS, spectral

range 170 to 2100 nm) coupled to an Acton SP2150 monochromator (600 g/nm, 300 nm Blaze) allowing excitation wavelengths to be selected with a 6 nm FWHM band-pass. The collection of the emitted light was facilitated (90° angle set up, 1 cm path length quartz cuvette) by a Lock-In Amplifier (Hinds Instruments Signaloc 2100) and Photoelastic Modulator (Hinds Series II/FS2AA). The differentiated light was focused onto an Acton SP2150 monochromator (1200 g/nm, 500 nm Blaze) equipped with ahigh sensitivity cooled Photo Multiplier Tube (Hamamatsu H10723-20 PhotoSensor red corrected). The detection of the CPL signal was achieved using the field modulation lock-in technique. The electronic signal from the PMT was fed into the lock-in amplifier (Hinds Instruments Signaloc 2100). The reference signal for the lock-in detection was provided by the PEM control unit. The monochromators, PEM control unit and lock-in amplifier were interfaced with a desktop PC and controlled by Labview code.

Gaussian 09 (Revision D.01) program was used for geometry optimization and frequency calculations.[1] Density functional theory (DFT) method M06-2X [2] with cc-pVDZ basis set[3] and Stuttgart ECP[4] for the lanthanide ion were employed. Solvent effects were accounted with using the Solvation Model based on Density (SMD) parameterisation for the continuum model[5] of water. An additional empirical dispersion correction GD3[6] was introduced to account for weak interactions. Real harmonic frequencies ensured that the optimized structures are local minima on their respective potential energy surfaces.

Proton NMR spectra were processed with the MestReNova v11.0.3 software to get positions, linewidths and integrals for all signals. Paramagnetic shifts (difference in chemical shifts between paramagnetic examples and diamagnetic yttrium(III) complexes) are dominated by the pseudocontact contribution (PCS) in the case of dysprosium(III). Therefore, it was fitted with the following expression:

$$\delta_{para} - \delta_{dia} = \frac{10^6}{4\pi r^5}\left[\chi_{xx}\left(x^2 - z^2\right) + \chi_{xy}\left(2xy\right) + \chi_{xz}\left(2xz\right) + \chi_{yy}\left(y^2 - z^2\right) + \chi_{yz}\left(2yz\right)\right] \quad (0.1)$$

where the shift is in the units of ppm, coordinates are defined relative to the lanthanide in Å, components of the traceless part of the susceptibility tensor are in the units of Å^3 in SI system. Atomic coordinates were taken from DFT optimized geometries, and for methyl groups the structural functions were averaged assuming their fast rotation. Since PCS depends linearly on five components of the susceptibility tensor, the robust linear regression with 'bisquare' weight function as implemented in MATLAB R2017a was used to fit their values. The permutation with the largest adjusted Pearson coefficient was taken as the best fit. Using the best-fit χ tensor, the PCS values for all other protons have been predicted. The line widths were fitted using a simple isotropic model:

$$\Delta\nu = \frac{C_1}{r^6} + C_2 \quad (0.2)$$

where the constants C_1 and C_2 were fitted for the assigned signals and used to predict linewidth for all other protons. We have used total shift *vs.* width plots of predicted and experimentally observed signals for further assignment. Well-isolated groups of experimental signals were assigned to closely located

groups of predicted signals and all possible permutations within the groups were checked, the assignment that gave the largest adjusted Pearson coefficient was chosen as the best fit.

The source code for this procedure will be released in *Spinach* version 2.2.[7]

The best-fit susceptibility tensors are presented in the main text as axiality, rhombicity and three Euler angles. We use the following convention for defining axiality, rhombicity and Euler angles of a traceless susceptibility tensor. Diagonalisation of a traceless susceptibility tensor gives eigenvalues and eigenvectors which are labelled to satisfy the relation $|\chi_x|<|\chi_y|<|\chi_z|$. Then axiality is defined as 3/2 χ_z and rhombicity as $(\chi_x - \chi_y)/2$. In such a definition, axiality and rhombicity have the same sign and the limiting value of the ratio of rhombicity to axiality is 1/3 ($0<\chi_{rh}/\chi_{ax}<1/3$).

Synthesis

The DO2A-ethyl ester[8] was synthesised according to previously reported procedures,.

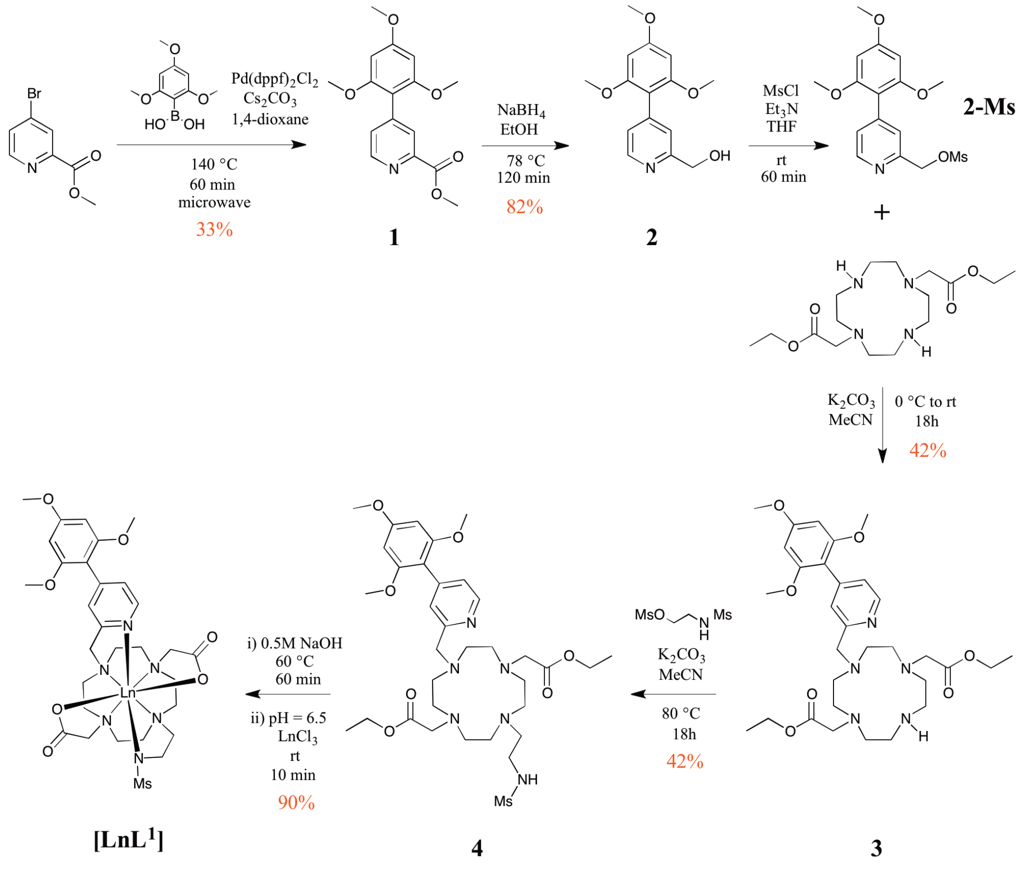

Scheme 1 Synthetic procedure for **[LnL^1]**

Methyl 4-(2,4,6-trimethoxyphenyl)picolinate,

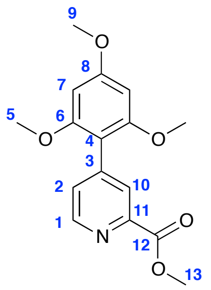

2,4,6-Trimethoxyphenyl boronic acid (200 mg, 0.94 mmol), methyl 4-bromopicolinate (160 mg, 0.74 mmol), caesium carbonate (350 mg, 1.07 mmol) and Pd(dppf)$_2$Cl$_2$ (20 mg, 0.027 mmol) were placed into a microwaveable vial, sealed, evacuated and refilled with argon. Dry 1,4-dioxane (1.5 mL) was added and two freeze-pump-thaw cycles were carried out to degas the solution. The reaction mixture was microwaved at 150 °C for 40 min. The crude product was purified on a silica column (0% to 5% of MeOH in DCM), giving a mixture of the desired product and 2,4,6-trimethoxybenzene as a side product. The fractions containing both compounds were combined and purified on RP-HPLC (10% to 100% acetonitrile (+0.1% formic acid) in water (+0.1% formic acid) over 10 min). The fractions containing the desired product were combined and neutralised with aqueous ammonia solution. The solvent was removed under reduced pressure, dried and re-dissolved in acetonitrile. Ammonium formate was filtered off and the solvent was removed under reduced pressure, giving an off-white solid (75 mg, 33% yield); ^{1}H NMR (295 K, 400 MHz, CDCl$_3$) δ_H 8.72 (1H, d, $^3J_{H-H}$ = 4.5 Hz, H^1), 8.18 (1H, m, H^{10}), 7.50 (1H, dd, $^3J_{H-H}$ = 4.5 Hz, $^4J_{H-H}$ = 1.5 Hz, H^2), 6.24 (2H, s, H^7), 4.03 (3H, s, H^{13}), 3.90 (3H, s, H^9), 3.76 (6H, s, H^5); ^{13}C NMR (295 K, 100 MHz, CDCl$_3$) 165.9 (C^{12}), 161.8 (C^8), 158.2 (C^6), 148.6 (C^1), 129.8 (C^2), 128.2 (C^{10}), 108.4 (C^4), 90.8 (C^7), 55.8 (C^5), 55.4 (C^9), 52.8 (C^{13}); *m/z* (HRMS$^+$) 304.1187 [M+H$^+$]$^+$ ($C_{16}H_{18}NO_5$ requires 304.1185).

(4-(2,4,6-Trimethoxyphenyl)pyridin-2-yl)methanol, 2

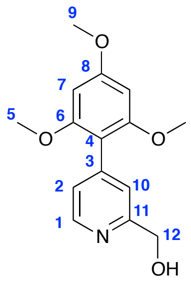

Methyl 4-(2,4,6-trimethoxyphenyl)picolinate, **1** (150 mg, 0.50 mmol) was dissolved in 5 mL of ethanol (200 proof) and $NaBH_4$ (130 mg, 3.42 mmol) was added. The reaction mixture was boiled under reflux at 78 °C for 2 h and the reaction mixture was quenched by adding water (35 mL). The solution was washed with DCM (3 x 40 mL), organic fractions were combined and dried over $MgSO_4$ and the solvent was removed under reduced pressure giving an off-white solid (112 mg, 82% yield). ^{1}H NMR (295 K, 400 MHz, $CDCl_3$) δ_H 8.55 (1H, $^{3}J_{H\text{-}H}$ = 5.0 Hz, H^{1}), 7.25 (1H, m, H^{10}), 7.23 (1H, dd, $^{3}J_{H\text{-}H}$ = 5.0 Hz, $^{4}J_{H\text{-}H}$ = 1.4 Hz), 6.24 (2H, s, H^{7}), 4.80 (2H, s, H^{12}), 3.89 (3H, s, H^{9}), 3.75 (6H, s, H^{5}); ^{13}C NMR (295 K, 100 MHz, $CDCl_3$) δ_C 161.6 (C^{8}), 158.2 (C^{6}), 158.0 (C^{11}), 147.5 (C^{1}), 143.7 (C^{3}), 125.4 (C^{2}), 123.4 (C^{10}), 109.4 (C^{4}), 90.9 (C^{7}), 64.2 (C^{12}), 55.8 (C^{5}), 55.4 (C^{9}); *m/z* (HRMS^{+}) 276.1245 $[M+H^{+}]^{+}$ ($C_{15}H_{18}NO_4$ requires 276.1236).

Diethyl 2,2'-(4-((4-(2,4,6-trimethoxyphenyl)pyridin-2-yl)methyl)-1,4,7,10-tetraazacyclododecane-1,7-diyl)diacetate, 3

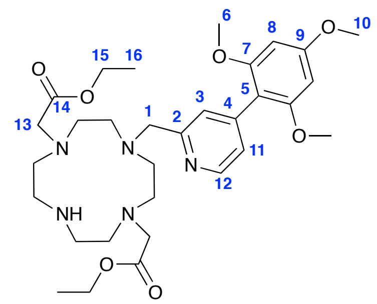

(4-(2,4,6-Trimethoxyphenyl)pyridin-2-yl)methanol, **2** (56 mg, 0.20 mmol) was dissolved in 5 mL of dry THF, followed by addition of 0.1 mL of dry triethylamine (0.72 mmol) and 0.045 mL of MsCl (0.58 mmol). The reaction mixture was stirred for 1 h at rt and the solvent was removed under reduced pressure. The crude material was dissolved in DCM (25 mL), washed with water (2 x 25 mL) and dried over $MgSO_4$. The solvent was removed under reduced pressure, dried and dissolved in dry acetonitrile (3 mL). DO2A-ethyl ester (100 mg, 0.29 mmol) was dissolved in dry acetonitrile (9 mL) and K_2CO_3 was added (150 mg, 1.09 mmol). The reaction mixture was cooled down using an ice bath and stirred for 5 minutes, followed by dropwise addition of the **2-Ms** within 5 min. The reaction mixture was allowed to reach rt and stirred for 18h. The reaction mixture was filtered and purified on RP-HPLC (10% to 100% acetonitrile (+0.1% formic acid) in water (+0.1% formic acid) over 10 min). The fractions containing the desired product were combined and neutralised with aqueous ammonia solution. The solvent was removed under reduced pressure, dried and redissolved in acetonitrile. Ammonium formate was filtered off and the solvent was removed under reduced pressure, giving a transparent oil (50 mg, 42% yield); ^{1}H NMR (295 K, 700 MHz, $CDCl_3$) δ_H 8.60 (1H, $^{3}J_{H\text{-}H}$ = 5.5 Hz, H^{12}), 7.19 (2H, m, H^{3}, H^{11}), 6.20 (2H, s, H^{8}), 4.11 (4H, t, $^{3}J_{H\text{-}H}$ = 7.0 Hz, H^{15}), 3.85 (3H, s, H^{10}), 3.72 (2H, m, H^{1}), 3.70 (6H, s, H^{6}), 3.23 (4H, s, H^{13}), 3.11-2.62 (16H, m, cyclen), 1.23 (6H, $^{3}J_{H\text{-}H}$ = 7.0 Hz, H^{16}); ^{13}C NMR (295 K, 176 MHz, $CDCl_3$) δ_C 171.4

(C^{14}), 161.6 (C^{9}), 158.0 (C^{7}), 156.2 (C^{2}), 148.7 (C^{12}), 143.5 (C^{4}), 126.8 (C^{11}), 125.5 (C^{3}), 109.3 (C^{5}), 90.8 (C^{8}), 60.5 (C^{15}), 57.1 (C^{1}), 55.7 (C^{6}), 55.6 (C^{13}), 55.4 (C^{10}), 54.7, 51.0, 50.5, 46.3 (cyclen), 14.2 (C^{16}); *m/z* ($HRMS^{+}$) 602.3557 $[M+H^{+}]^{+}$ ($C_{31}H_{48}N_5O_7$ requires 602.3554).

Diethyl 2,2'-(4-(2-(methylsulfonamido)ethyl)-10-((4-(2,4,6-trimethoxyphenyl)pyridin-2-yl)methyl)-1,4,7,10-tetraazacyclododecane-1,7-diyl)diacetate, 4

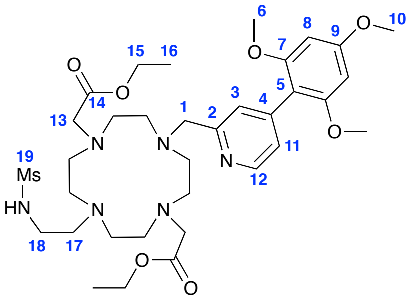

Diethyl 2,2'-(4-((4-(2,4,6-trimethoxyphenyl)pyridin-2-yl)methyl)-1,4,7,10-tetraazacyclo-dodecane-1,7-diyl)diacetate, **3** (50 mg, 0.083 mmol) was dissolved in dry acetonitrile (5 mL), followed by addition of K_2CO_3 (28 mg, 0.20 mmol) and 2-(methylsulfonamido)ethyl methanesulfonate (18 mg, 0.083 mmol). The reaction mixture was immersed into an oil bath and heated up initially to 80 °C and stirred at this temperature for 18 h. The reaction mixture was filtered and purified using RP-HPLC (10% to 100% acetonitrile (+0.1% formic acid) in water (+0.1% formic acid) over 10 min). The fractions containing the desired product were combined and neutralised with aqueous ammonia solution. The solvent was removed under reduced pressure, dried and re-dissolved in acetonitrile. Ammonium formate was filtered off and the solvent was removed under reduced pressure, giving a transparent oil (25 mg, 42% yield); ^{1}H NMR (295 K, 700 MHz, $CDCl_3$) δ_H 8.53 (1H, $^{3}J_{H-H}$ = 5.0 Hz, H^{12}), 7.30 (1H, m, H^{3}), 7.27 (1H, dd, $^{3}J_{H-H}$ = 5.0 Hz, $^{4}J_{H-H}$ = 1.5 Hz, H^{11}), 6.19 (2H, s, H^{8}), 4.14 (2H, m, H^{1}), 4.13 (4H, q, $^{3}J_{H-H}$ = 7.0 Hz, H^{15}), 3.85 (3H, s, H^{10}), 3.72 (6H, s, H^{6}), 3.48 (2H, m, H^{18}), 3.40 (4H, s, H^{13}), 3.30 (2H, m, H^{17}), 3.22 – 2.92 (16H, m, cyclen), 2.96 (3H, s, H^{19}), 1.24 (6H, t, $^{3}J_{H-H}$ = 7.0 Hz, H^{16}); ^{13}C NMR (295 K, 176 MHz, $CDCl_3$) δ_C 170.7 (C^{14}), 166.6 (C^{9}), 161.8 (C^{7}), 153.1 (C^{2}), 148.7 (C^{12}), 144.0 (C^{4}), 128.0 (C^{3}), 126.2 (C^{11}), 90.9 (C^{8}), 108.6 (C^{6}), 60.8 (C^{15}), 59.1 (C^{1}), 56.0 (C^{13}), 55.8 (C^{6}), 55.4 (C^{10}), 53.4 (C^{18}), 53.1, 52.8, 49.9, 49.8 (cyclen), 39.1 (C^{19}), 38.7 (C^{17}), 14.1 (C^{16}); *m/z* ($HRMS^{+}$) 723.3771 $[M+H^{+}]^{+}$ ($C_{34}H_{55}N_6O_9S$ requires 723.3751).

[LnL[5]] (Ln = Eu, Tb, Dy, Y)

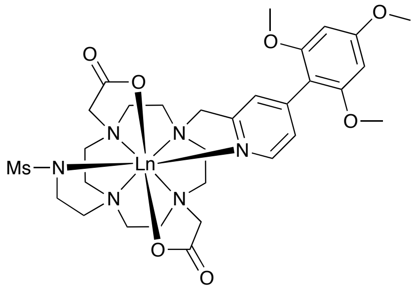

The ligand, **4**, (0.020 mmol) was dissolved in aqueous NaOH solution (0.5 M, 4 mL) and stirred at 60 °C for 2 h. The solution was neutralised by addition of 1 M HCl and $LnCl_3$ (0.030 mmol) was added. The reaction mixture was stirred at rt for 1 h and pH was adjusted to 6.5. The reaction mixture was stirred for another hour and a desired complex was purified on RP-HPLC (Chromolith® column, 0% to 100% acetonitrile in water). The combined fractions were freeze-dried to give an off-white solid in quantitative yield.

[EuL^5]: *m/z* (HRMS$^+$) 815.2113 [M+H$^+$]$^+$ ($C_{30}H_{44}N_6O_9S^{151}Eu$ requires 815.2089).

[TbL^5]: *m/z* (HRMS$^+$) 823.2137 [M+H$^+$]$^+$ ($C_{30}H_{44}N_6O_9S^{159}Tb$ requires 823.2144); $\varepsilon(H_2O)$ = 12800 $M^{-1}cm^{-1}$; $\tau(H_2O)$ = 1.14 ms (pH = 8), $\tau(D_2O)$ = 1.92 ms (pD = 8.4); n.b. Tb has only one abundant isotope.

[DyL^5]: *m/z* (HRMS$^+$) 824.2172 [M+H$^+$]$^+$ ($C_{30}H_{44}N_6O_9S^{160}Dy$ requires 824.2143).

[YL^5]: *m/z* (HRMS$^+$) 753.1941 [M+H$^+$]$^+$ ($C_{30}H_{44}N_6O_9S^{89}Y$ requires 753.1949).

Figures and Tables

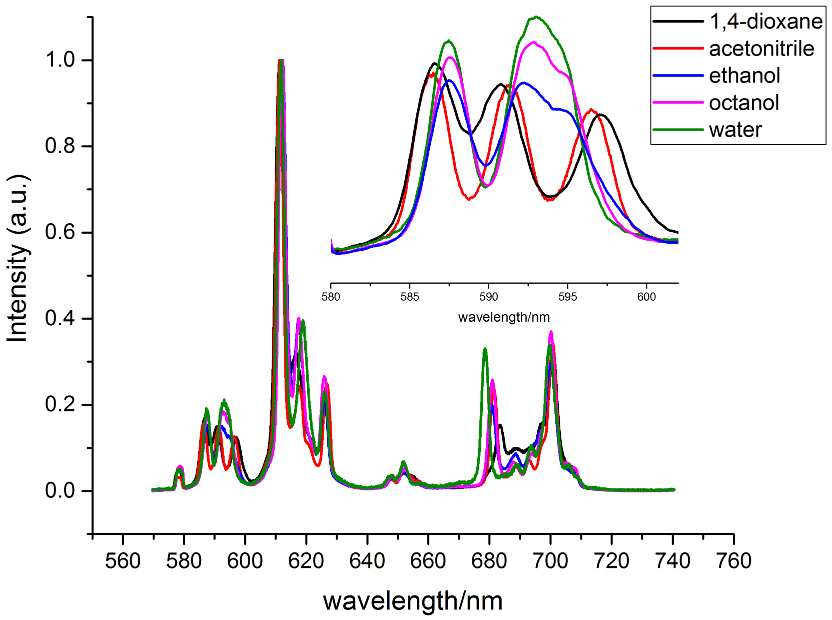

Fig. S1 Emission spectra of **[EuL[1]]** in different organic solvents and in water, with a magnified region for the $^5D_0 \rightarrow {}^7F_1$ manifold (298 K, λ_{ex} = 390 nm).

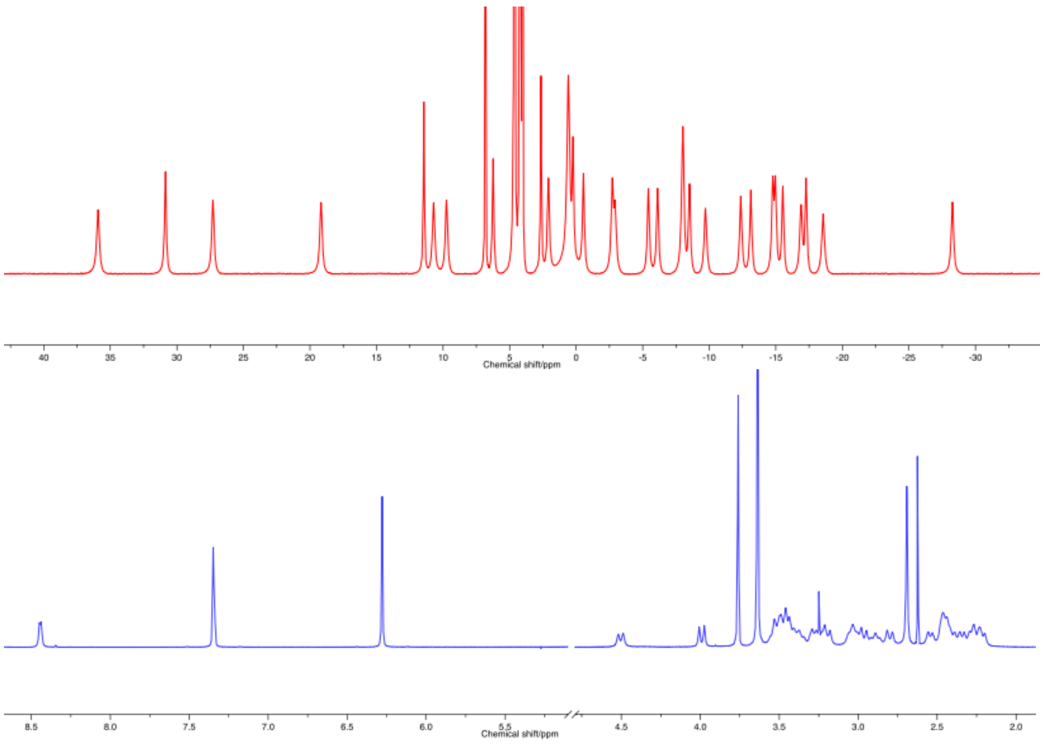

Fig. S2 ^1H NMR spectra of **[EuL[1]]** (*top*) and **[YL[1]]** (*bottom*) (D_2O, 295 K, 9.4 T for **[EuL[1]]** and 278 K, 11.7 T for **[YL[1]]**).

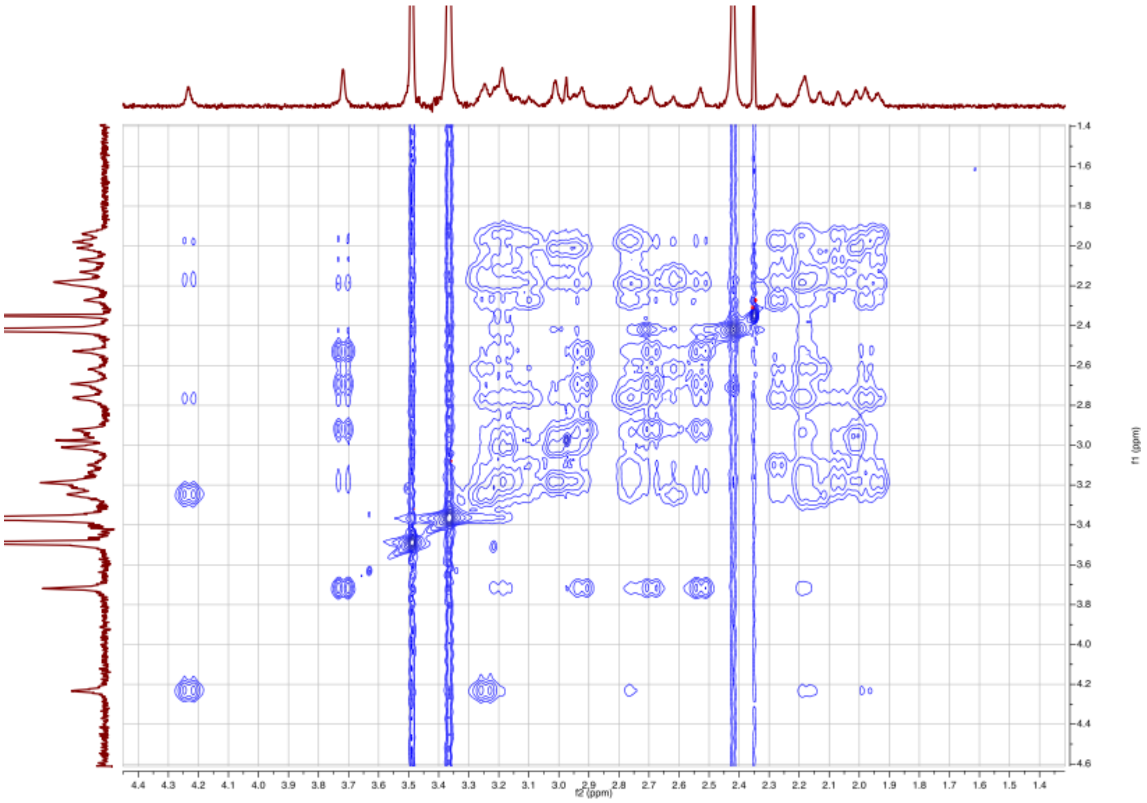

Fig. S3 ^1H-^1H NOESY spectrum of **[YL^1]** (D_2O, 278 K, 11.7 T).

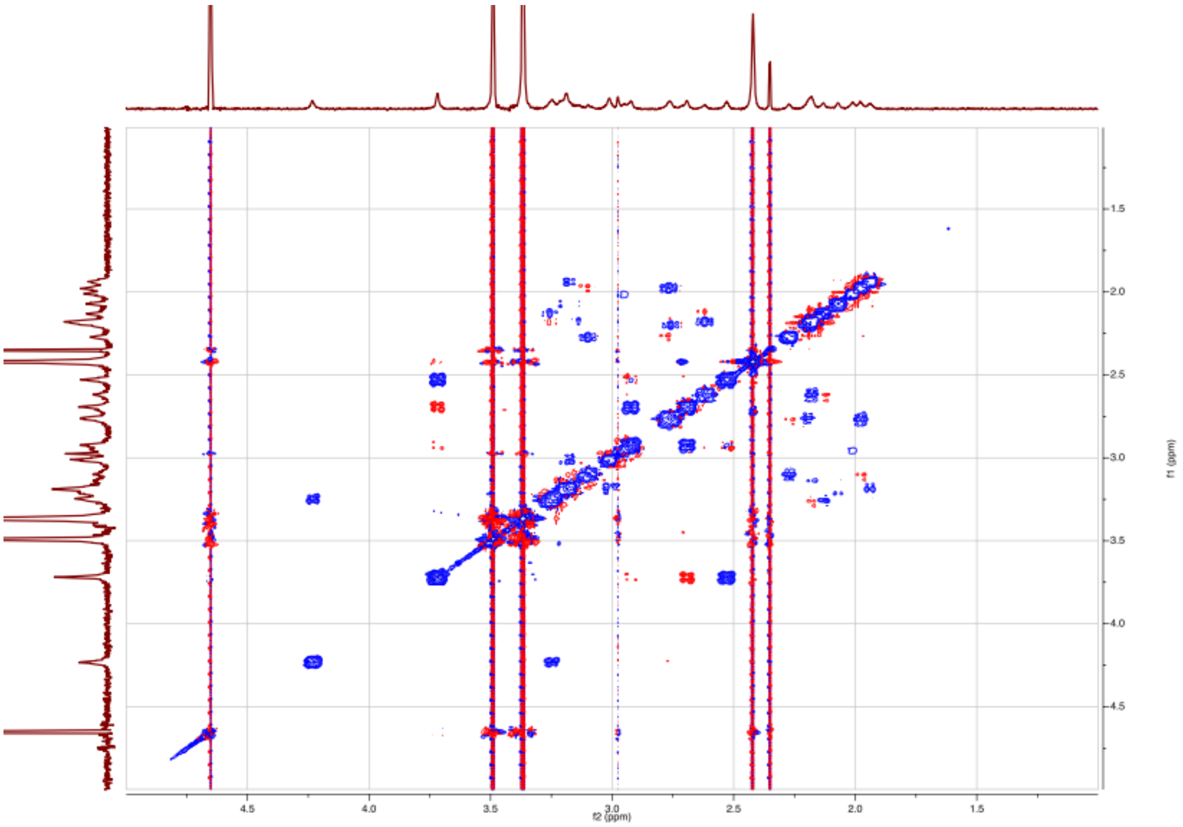

Fig. S4 ^1H-^1H ROESY spectrum of **[YL^1]** (D_2O, 278 K, 11.7 T).

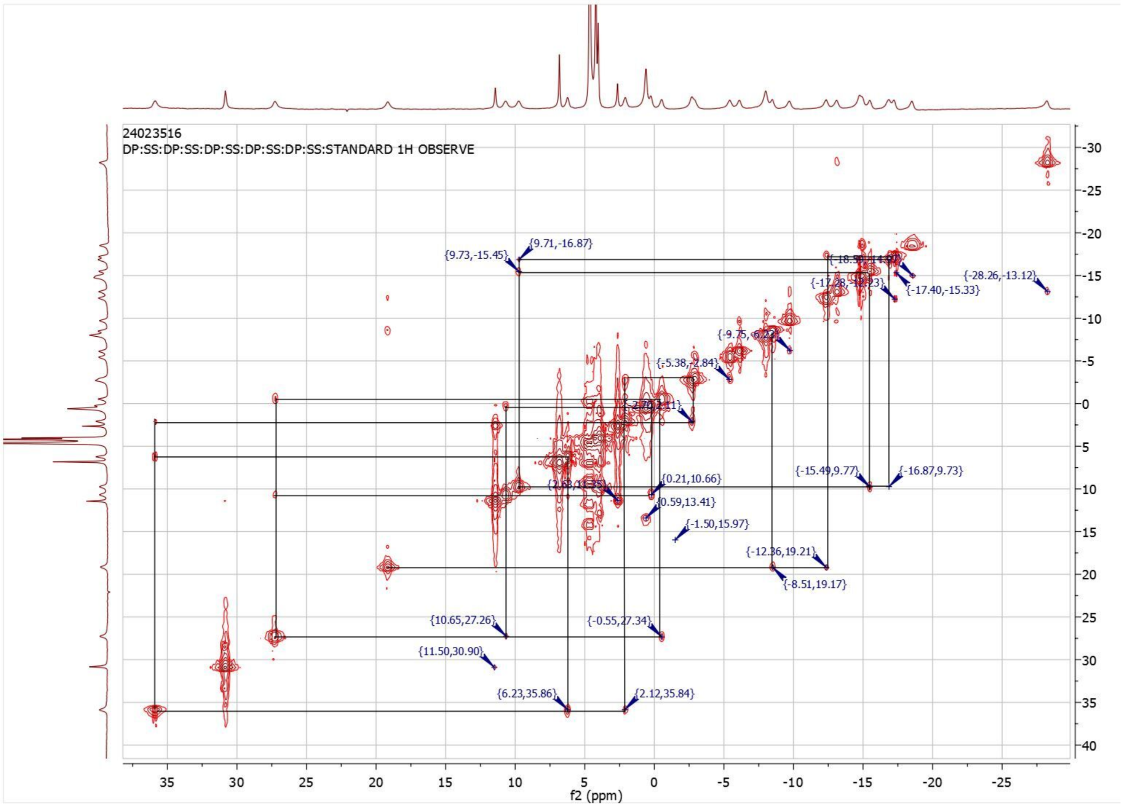

Fig. S5 ^{1}H-^{1}H COSY spectrum of **[EuL^{1}]** (D_2O, 278 K, 9.4 T).

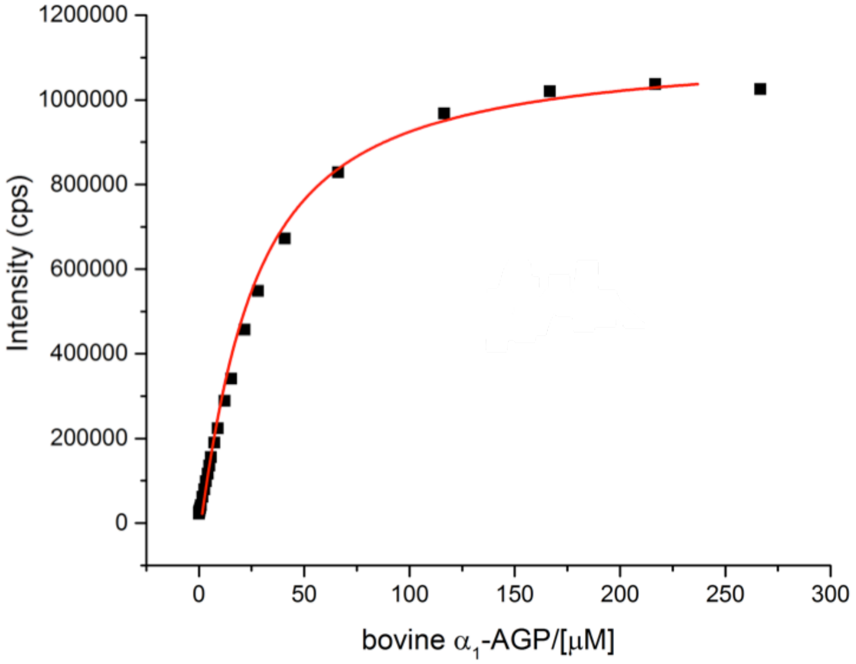

Fig. S6 Change of the total emission intensity upon addition of bovine α_1-AGP to **[EuL^{1}]** (**[EuL^{1}]** 11 μM; log K = 4.7(01), assuming a 1:1 binding isotherm, λ_{ex} = 310 nm, 298 K, 0.1 M HEPES, pH = 7.40).

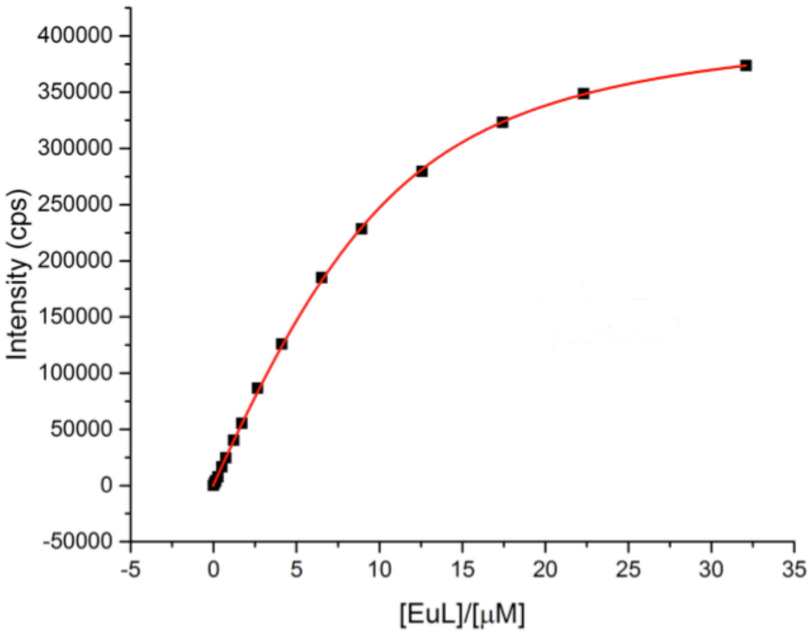

Fig. S7 Change of the total emission intensity upon addition of **[EuL[1]]** to bovine α_1-AGP ([bovine α_1-AGP] 25 µM; log K = 5.5 (01), assuming a 1:1 binding isotherm, λ_{ex} = 310 nm, 298 K, 0.1 M HEPES, pH = 7.40).

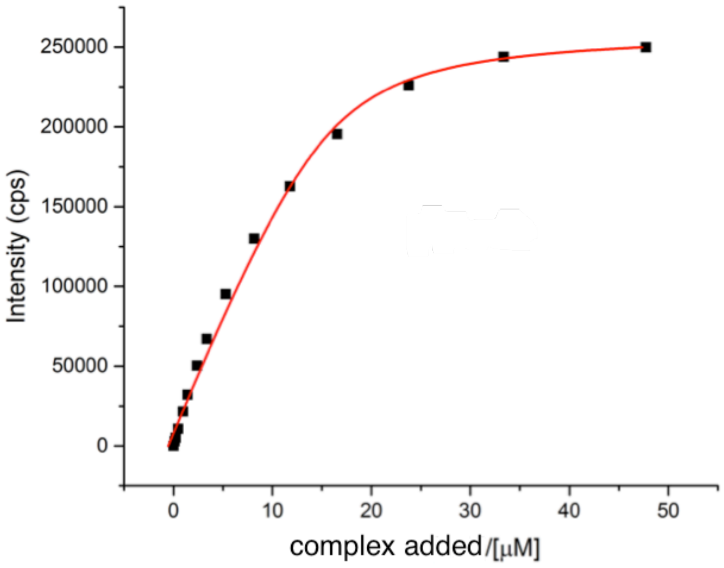

Fig. S8 Change of the total emission intensity upon addition of **[EuL[1]]** to human α_1-AGP ([human α_1-AGP] 25 µM; log K = 5.85(0.1), assuming a 1:1 binding isotherm, λ_{ex} = 310 nm, 298 K, 0.1 M HEPES, pH = 7.40).

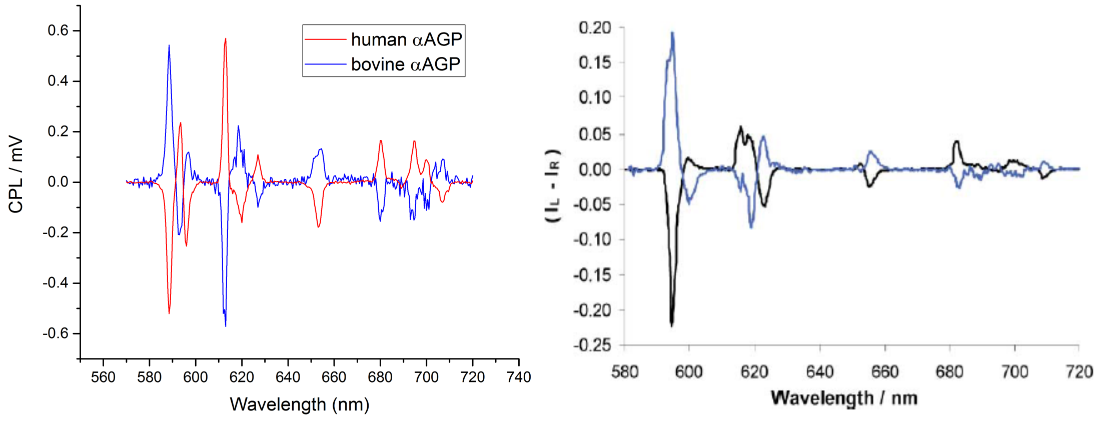

Fig. S9 Comparison between CPL spectra recorded for **[EuL^{1}]** in the presence of human and bovine α_1-AGP at pH = 9.30 (*left*) and CPL spectra of serum-albumin bound Eu tetraazatriphenylene complexes reported earlier [10]; here the *Δ(δδδδ)* **[EuL^{1}]** enantiomer (*blue/blue)* is hypothesised to bind prefentially with added bovine α_1-AGP, and the *Λ(λλλλ)* enantiomer (*black/red*) may be preferentially bound when **[EuL^{1}]** is added to human α_1-AGP.

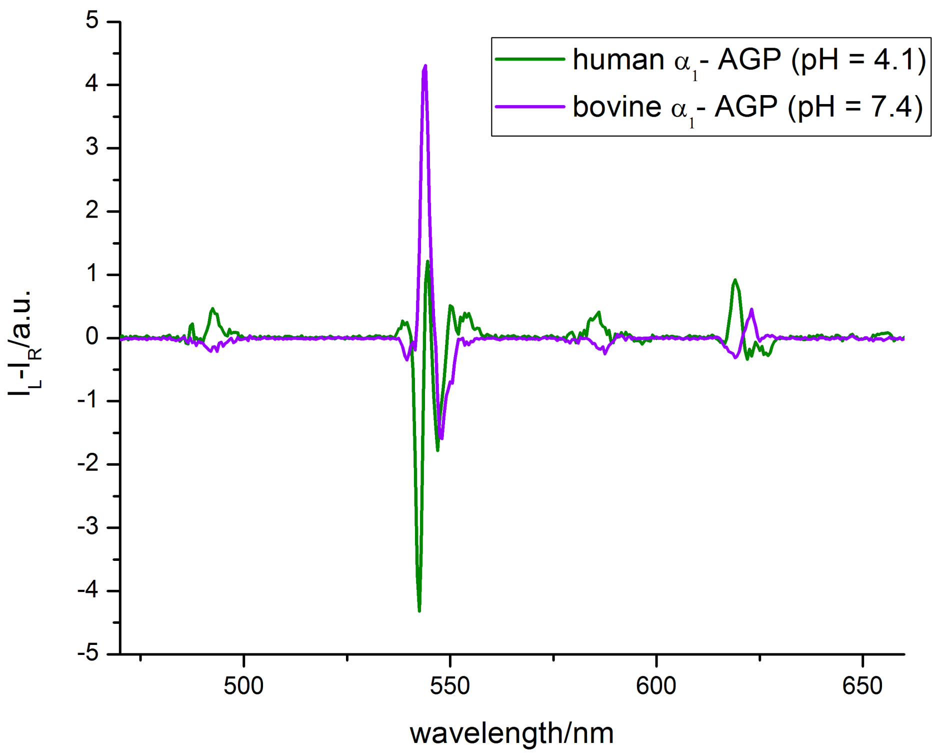

Fig. S10 Induced CPL spectra for **[TbL^{1}]** (8 μM) bound with human (green) and bovine (purple) α_1-AGP; the sulfonamide arm is not coordinated at lower pH (λ_{ex} = 310 nm, 0.1 M HEPES, pH = 4.1 or 7.40, 298 K).

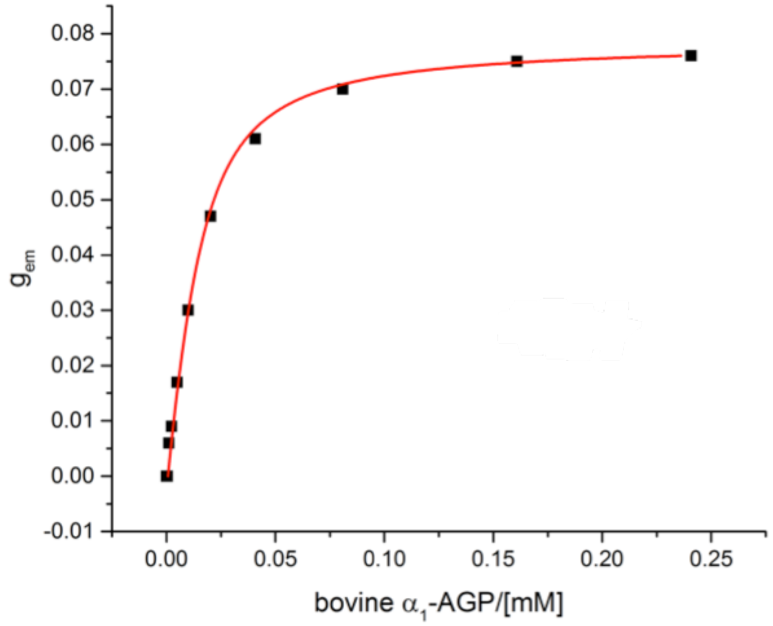

Fig. S11 Change of the emission dissymmetry factor upon addition of bovine α_1-AGP to **[TbL[1]]** (**[TbL[1]]** 8 μM; log K = 5.1(01), assuming a 1:1 binding isotherm, λ_{ex} = 310 nm, λ_{em} = 545 nm, 298 K, 0.1 M HEPES, pH = 7.40).

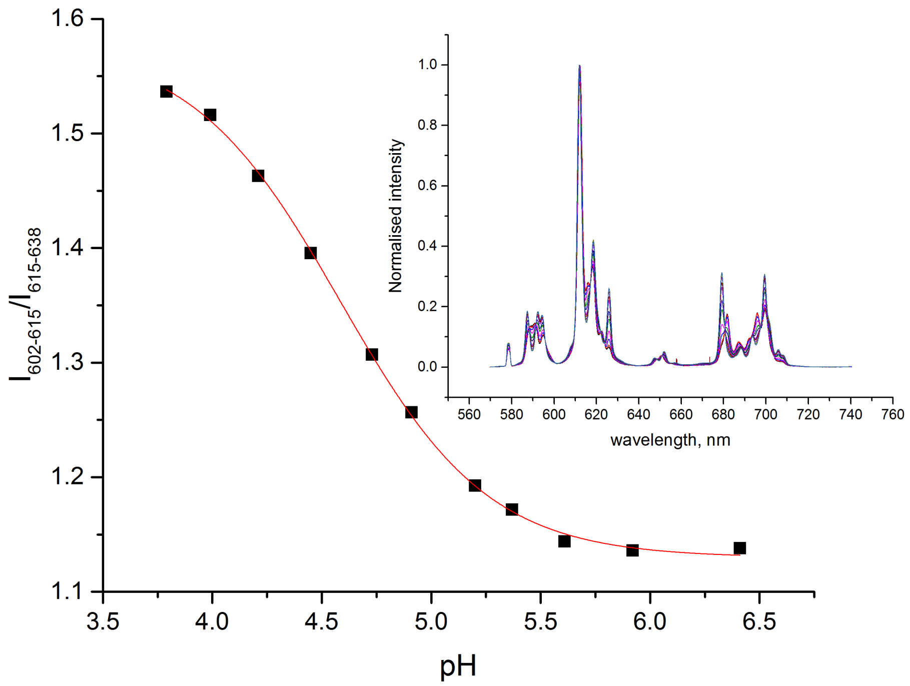

Fig. S12 pH calibration curves for **[EuL[1]]** (6 μM) following the relative intensities of two bands (602-615 nm vs. 615-638 nm) of the $^5D_0 \rightarrow ^7F_2$ transition manifold in the emission spectrum with human α_1-AGP (125 μM human α_1-AGP , λ_{ex} = 310 nm, 298 K), τ(pH = 3.79) = 0.91 ms, τ(pH = 6.41) = 0.78 ms

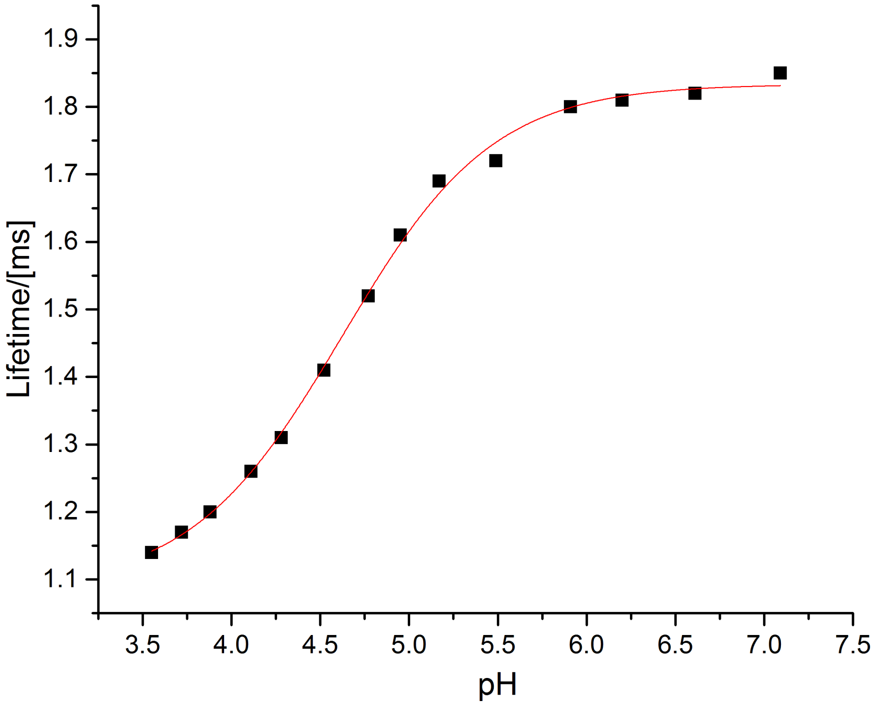

Fig. S13 pH calibration curve for **[TbL[1]]** (5 μM) following changes in the lifetime of the Tb $^{5}D_{4}$ excited state (0.1M NaCl, λ_{ex} = 310 nm, 298 K).

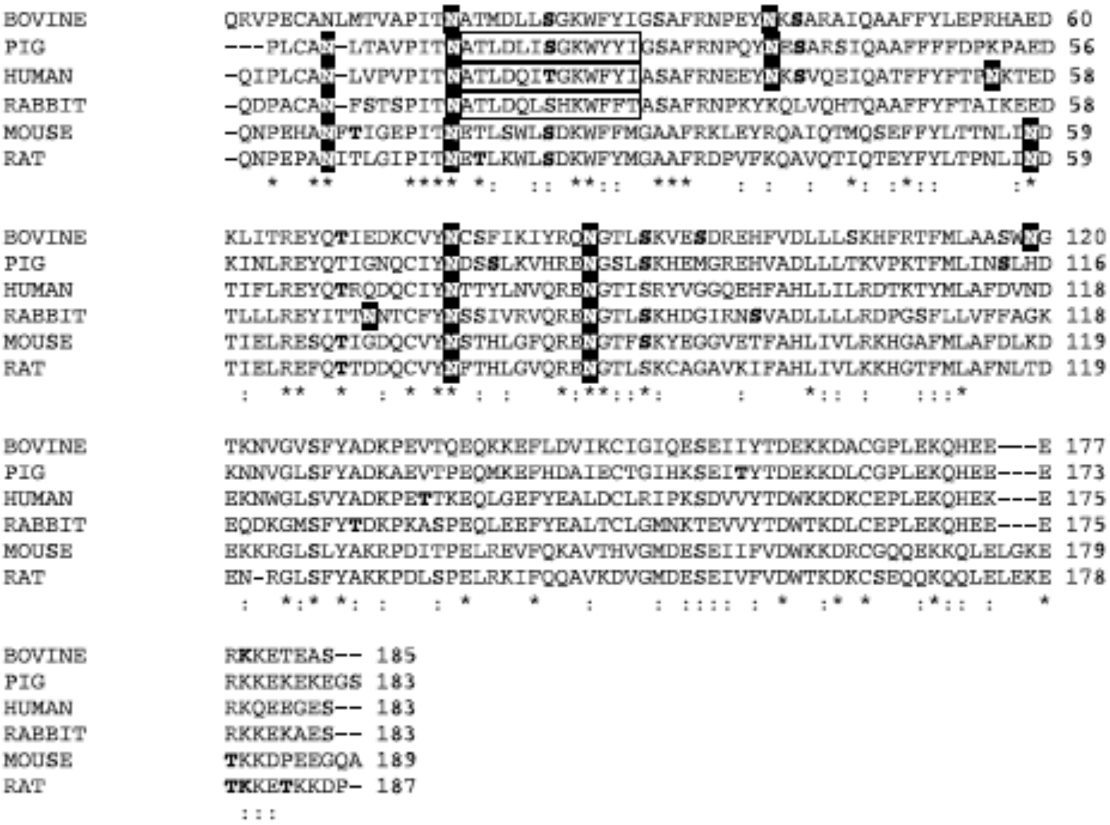

Fig. S14 Alignment of the sequences of various forms of α_1-AGP (F. Ceciliaini et al, *Vet. Res*. 2005, **36**, 735-746)

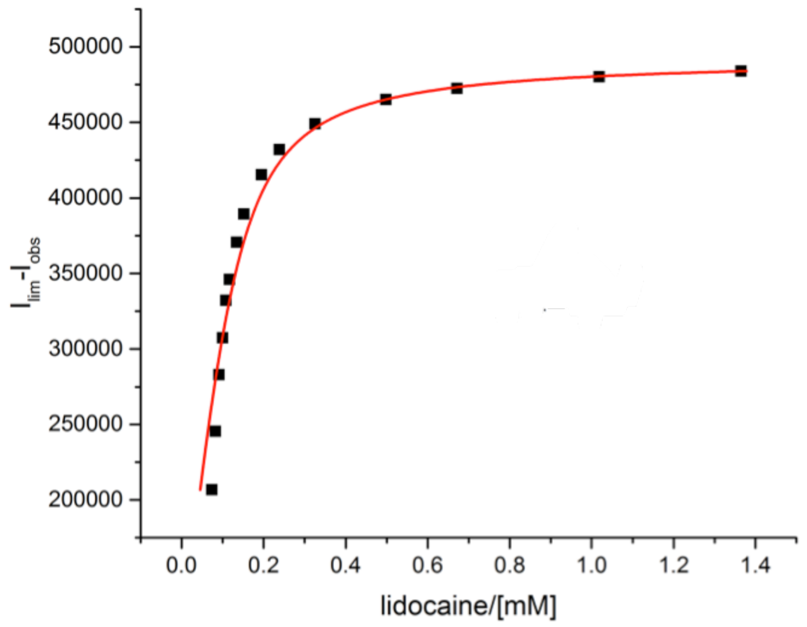

Fig. S15 Variation of europium emission intensity in the presence of human α_1-AGP showing the fit (*line*) to the data points with added lidocaine (**[EuL¹]** 3 µM; [human α_1-AGP] 122 µM, log K = 4.5(01), assuming a 1:1 binding isotherm, pH = 7.40, λ_{ex} = 310 nm, 0.1 M HEPES, 298 K).

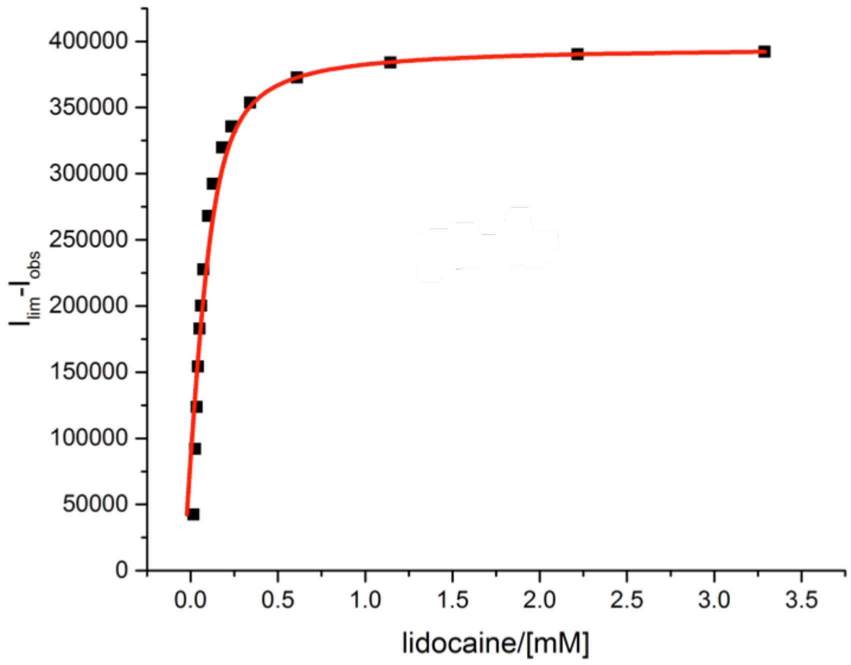

Fig. S16 Variation of europium emission intensity in the presence of bovine α_1-AGP showing the fit (*line*) to the data points with added lidocaine (**[EuL¹]** 3 µM; [bovine α_1-AGP] 122 µM, log K = 4.4(01), assuming a 1:1 binding isotherm, pH = 7.40, λ_{ex} = 310 nm, 0.1 M HEPES, 298 K).

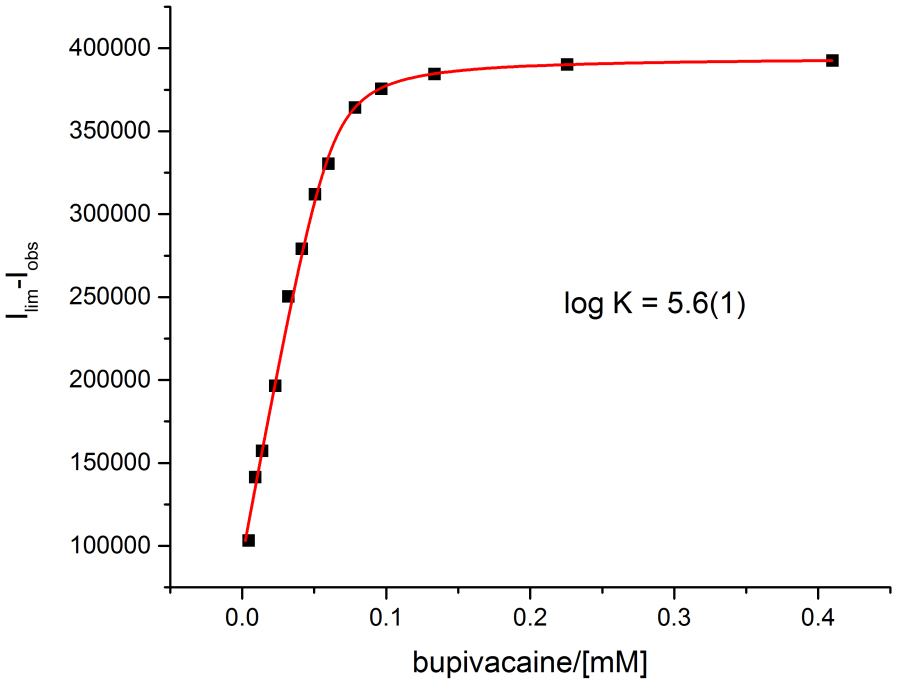

Fig. S17 Variation of europium emission intensity in the presence of human α_1-AGP showing the fit (*line*) to the data points with added bupivacaine (**[EuL[1]]** 3 μM; [human α_1-AGP] 135 μM, log K = 5.6(01), assuming a 1:1 binding isotherm, pH = 7.40, λ_{ex} = 310 nm, 0.1 M HEPES, 298 K).

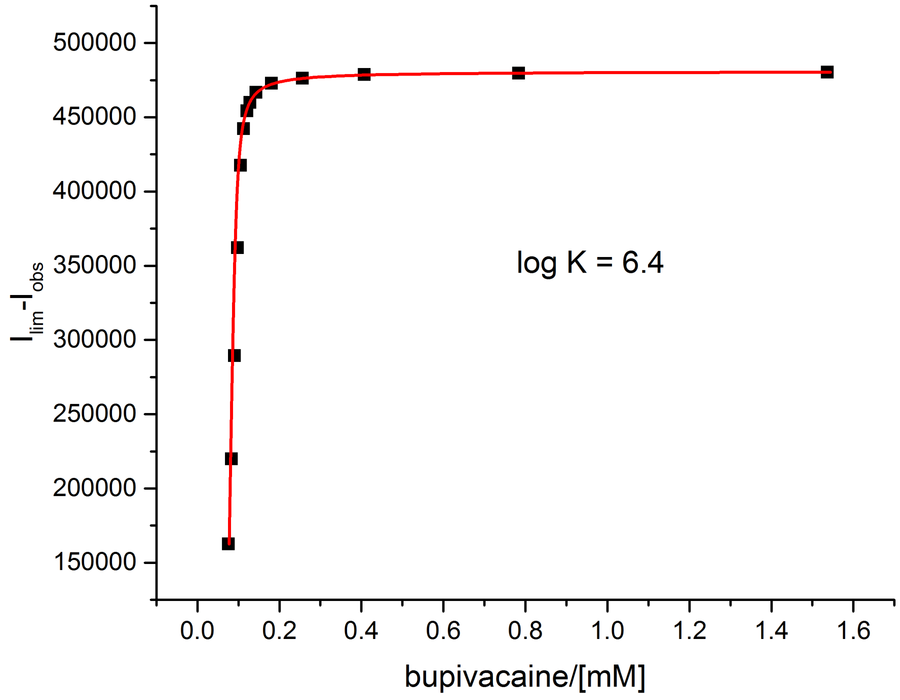

Fig. S18 Variation of europium emission intensity in the presence of bovine α_1-AGP showing the fit (*line*) to the data points with added bupivacaine (**[EuL[1]]** 3 μM; [bovine α_1-AGP] 122.0 μM, log K = 6.4, assuming a 1:1 binding isotherm, pH = 7.40, λ_{ex} = 310 nm, 0.1 M HEPES, 298 K).

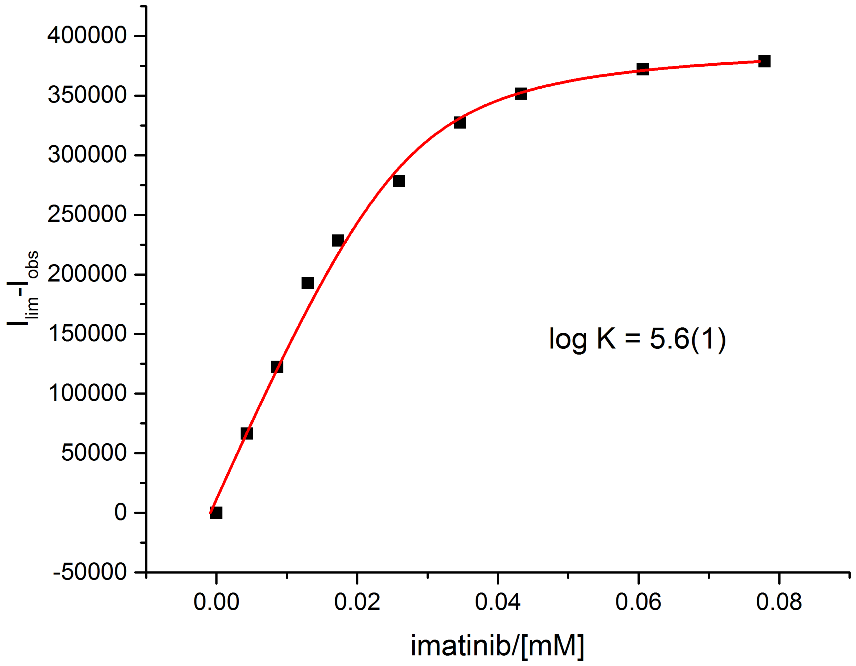

Fig. S19 Variation of europium emission intensity in the presence of human α_1-AGP showing the fit (*line*) to the data points with added imatinib (**[EuL¹]** 3 µM; [human α_1-AGP] 135.0 µM, log K = 5.6(1), assuming a 1:1 binding isotherm, pH = 7.40, λ_{ex} = 310 nm, 0.1 M HEPES, 298 K).

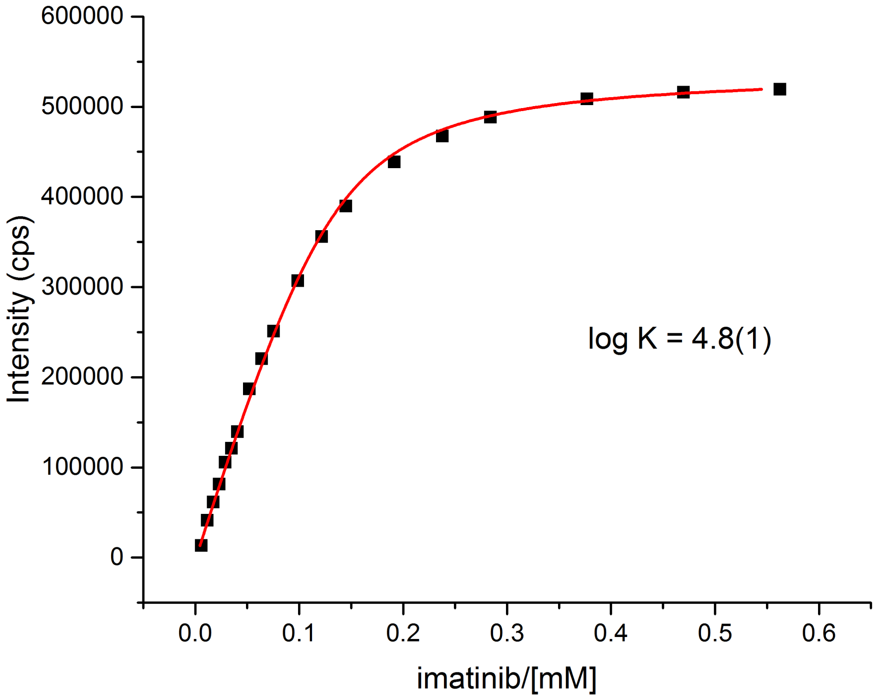

Fig. S20 Variation of europium emission intensity in the presence of bovine α_1-AGP showing the fit (*line*) to the data points with added imatinib (**[EuL¹]** 3 µM; [bovine α_1-AGP] 122 µM, log K = 4.8(1), assuming a 1:1 binding isotherm, pH = 7.40, λ_{ex} = 310 nm, 0.1 M HEPES, 298 K).

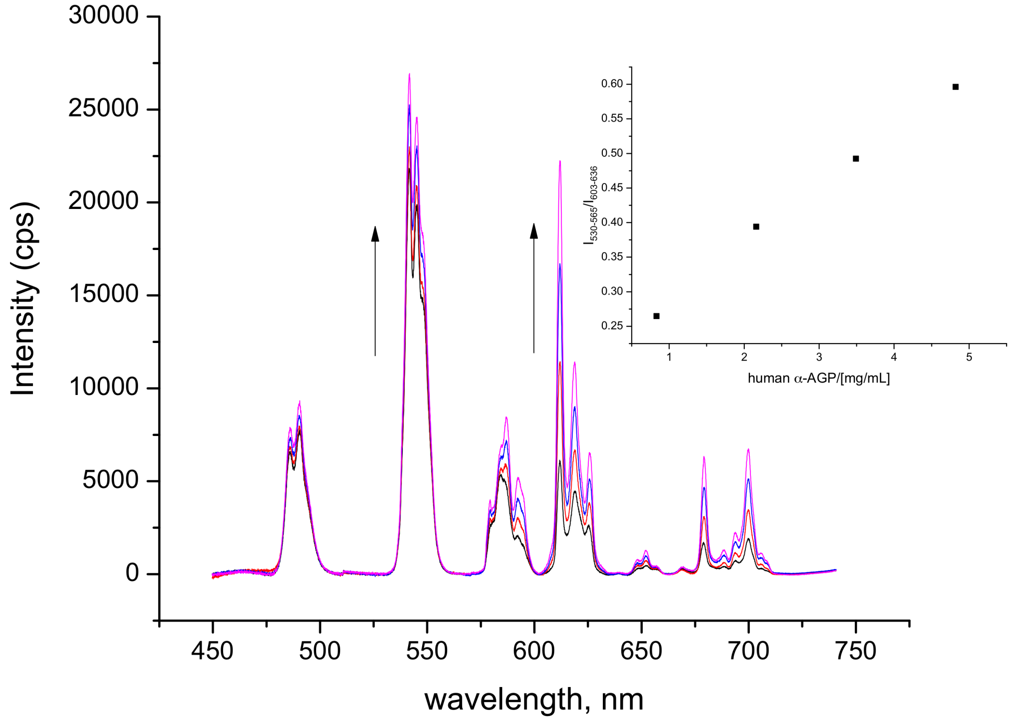

Fig. S21 Change of the total emission spectrum of **[EuL[1]]**/**[TbL[1]]** (22 μM/2 μM) 'cocktail' upon addition of human α_1-AGP to human serum (pH = 7.40, λ_{ex} = 310 nm, 298 K).

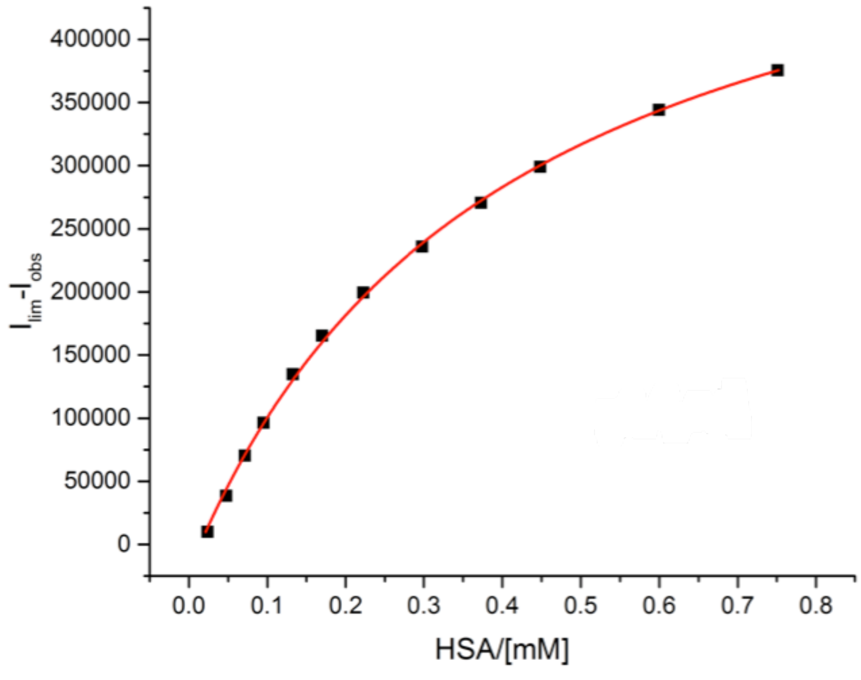

Fig. S22 Quenching of the total emission intensity of **[TbL[1]]** (8 μM) upon addition of HSA. The apparent binding constant is logK = 3.4(01) (pH = 7.40, 0.1 M HEPES, λ_{ex} = 310 nm, 298 K).

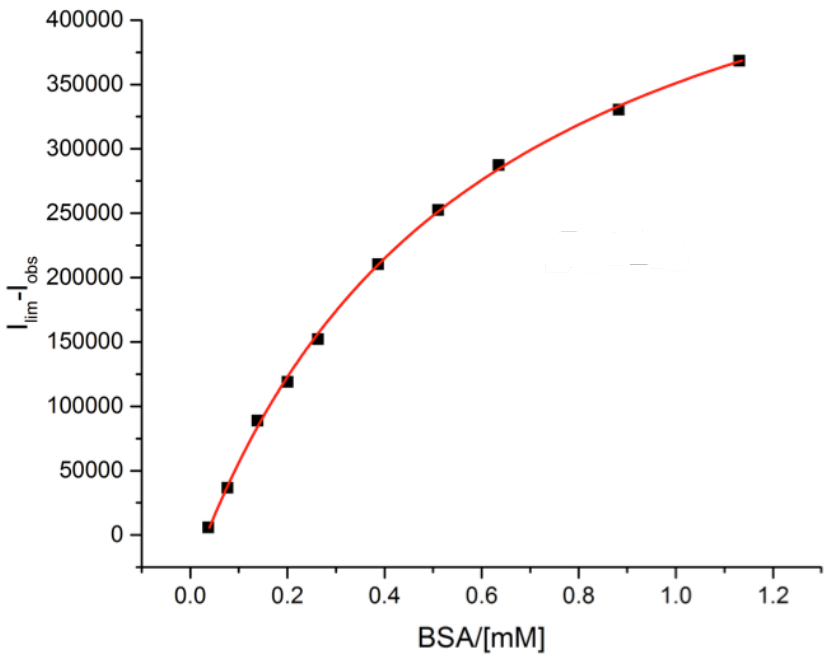

Fig. S23 Quenching of the total emission intensity of **[TbL[1]]** (8 μM) upon addition of BSA. The apparent binding constant is logK = 3.2(01) (pH = 7.40, 0.1 M HEPES, λ_{ex} = 310 nm, 298 K).

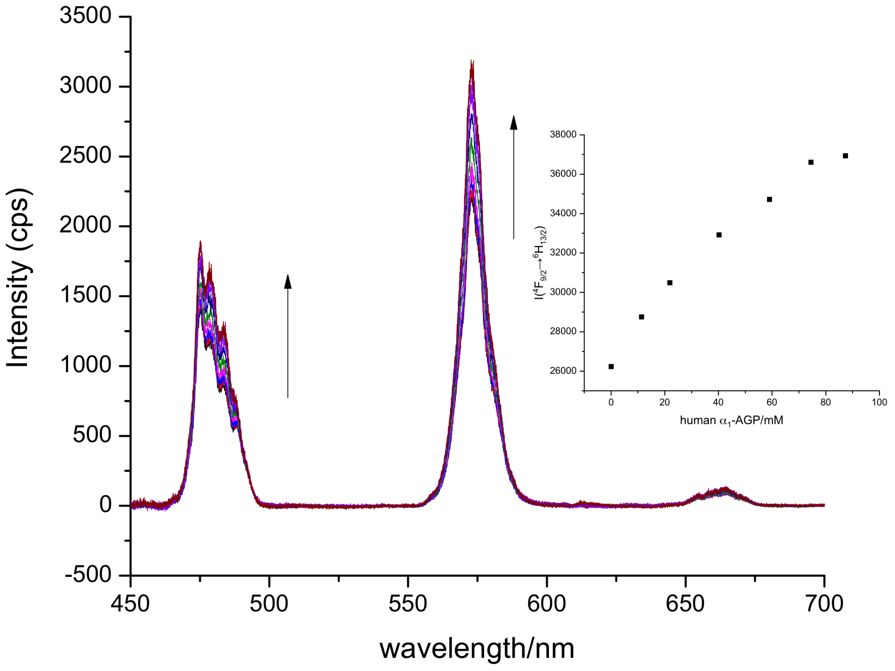

Fig. S24 Variation of the total emission intensity of **[DyL[1]]** (6 μM) as a function of added human α_1-AGP (pH = 7.40, 0.1 M HEPES, λ_{ex} = 310 nm, 298 K).

Table 1 Summary of the ^{1}H NMR solution data (D_2O, 300K, 43 MHz) and the best fitted values

Label	Diamagnetic shift, ppm	Experimental PCS, ppm	Theoretical PCS, ppm	Experimental width, Hz	Theoretical width, Hz	Averaged r^{-6}, $Å^{-6}$
MeS	2.61	-5.2	-4.9	50	168	3.937E-05
Meo	3.49	-28.4	-7.5	290	109	3.179E-07
Mep	3.37	-12.1	-15.0	68	112	2.555E-06
a1ax	3.50	204.8	215.4	889	971	5.652E-04
a1eq	2.50	179.6	182.3	399	322	1.400E-04
a3ax	3.50	204.8	202.3	889	731	4.081E-04
a3eq	2.50	149.9	164.4	269	312	1.337E-04
apax	4.23	400.4	390.1	1305	895	5.158E-04
apeq	3.25	121.9	125.1	334	303	1.278E-04
asax	3.50	297.3	309.2	1066	1100	6.496E-04
aseq	2.50	-40.0	-21.1	320	326	1.428E-04
aseq’	2.50	104.3	114.0	419	344	1.543E-04
c1ax	3.50	-211.6	-202.8	514	631	3.424E-04
c1eq	2.50	43.5	70.3	401	291	1.197E-04
c2ax	3.50	428.0	428.1	1200	703	3.896E-04
c2eq	2.50	53.4	77.5	381	293	1.211E-04
c3ax	3.50	-407.5	-402.4	486	609	3.279E-04
c3eq	2.50	-131.2	-111.8	265	290	1.189E-04
c4ax	3.50	-167.4	-164.7	575	688	3.801E-04
c4eq	2.50	-263.7	-233.3	249	298	1.243E-04
c5ax	3.50	-512.6	-523.8	420	674	3.705E-04
c5eq	2.50	-194.9	-183.8	284	311	1.331E-04
c6ax	3.50	224.3	236.7	948	797	4.512E-04
c6eq	2.50	-70.3	-48.3	273	305	1.290E-04
c7ax	3.50	-329.3	-327.0	464	636	3.460E-04
c7eq	2.50	-76.8	-45.6	285	288	1.180E-04
c8ax	3.50	75.3	75.5	550	670	3.682E-04
c8eq	2.50	-29.9	-5. 7	330	288	1.176E-04
ph3	6.01	-10.8	-10.5	60	110	1.151E-06
py3	7.07	9.4	10.9	168	160	3.410E-05
py5	7.07	-112.9	-108.2	141	158	3.245E-05
py6	8.17	-456.2	-528.8	835	1094	6.460E-04

Molecular frames of optimized geometries have been unified so that the origin is located at the lanthanide position, the z-axis is pointing into the middle of the four nitrogen atoms of the cyclen ring, and the x-axis is pointing at the first nitrogen atom of the cyclen, located under the pyridine.

Table 2 Optimized structures of **[LnL^{1}]** in the eight coordinate twisted square antiprismatic coordination geometry (TSAP), that defines the structure of the sulphonamide bound form that is observed in solution at higher pH values.

	Y			Eu			Tb			Dy		
Ln	0.0000	0.0000	0.0000	0.0000	0.0000	0.0000	0.0000	0.0000	0.0000	0.0000	0.0000	0.0000
S	-2.0629	-1.5258	-2.4693	-1.9988	-1.6043	-2.4543	-1.9470	-1.5652	-2.6001	-1.9585	-1.5245	-2.6087
O	0.7438	-4.1680	-1.1805	0.7997	-4.2214	-1.0769	0.8610	-4.2019	-1.1097	0.8196	-4.1868	-1.1369
O	0.9491	-1.9605	-0.8809	1.0045	-2.0080	-0.8086	0.9466	-1.9699	-0.8503	0.9114	-1.9585	-0.8542
O	-0.2944	4.2436	-1.1875	-0.2887	4.2636	-1.2691	-0.3103	4.2660	-1.2858	-0.2739	4.2317	-1.3116
O	-0.5806	2.0386	-0.9564	-0.5801	2.0585	-1.0336	-0.5780	2.0504	-1.0181	-0.5598	2.0237	-1.0105

O	-2.2706	-2.9790	-2.1463	-2.2186	-3.0564	-2.1389	-2.2070	-3.0227	-2.2699	-2.2255	-2.9860	-2.3017
O	-0.8033	-1.2275	-3.2263	-0.7119	-1.2963	-3.1550	-0.6509	-1.2868	-3.3201	-0.6623	-1.2402	-3.3271
O	5.9766	3.6742	-0.0929	6.3821	3.2004	-0.1942	6.2930	3.2859	-0.4694	6.2608	3.2993	-0.5334
O	5.7752	1.7128	-4.3423	5.6859	1.4765	-4.4999	5.4977	1.3413	-4.6855	5.4971	1.2690	-4.7141
O	9.4429	4.6508	-3.1830	9.7508	3.8829	-3.4602	9.4450	4.0814	-3.9481	9.4320	4.0359	-4.0083
N	2.0934	0.0000	1.6720	2.0994	0.0000	1.6717	2.1159	0.0000	1.6674	2.1142	0.0000	1.6559
N	-0.0233	-2.0824	1.6126	-0.0213	-2.0800	1.6642	-0.0362	-2.1029	1.6481	-0.0336	-2.1007	1.6480
N	-2.1034	-0.0085	1.6023	-2.1140	-0.0305	1.5720	-2.1158	-0.0287	1.5310	-2.1167	-0.0259	1.5402
N	0.0333	2.0908	1.6826	0.0360	2.1105	1.6046	0.0361	2.1316	1.6145	0.0360	2.1266	1.6156
N	2.2358	0.8225	-1.0346	2.2756	0.7874	-1.0357	2.2208	0.7071	-1.0737	2.2012	0.6924	-1.1039
N	-2.0933	-0.5687	-1.1815	-2.0777	-0.6501	-1.1711	-2.0152	-0.5831	-1.3130	-2.0185	-0.5624	-1.3069
C	0.5179	-3.1267	-0.5531	0.5693	-3.1686	-0.4712	0.5697	-3.1554	-0.5073	0.5399	-3.1479	-0.5173
C	-0.2132	3.1947	-0.5362	-0.2169	3.2155	-0.6161	-0.2210	3.2211	-0.6177	-0.1991	3.1987	-0.6247
C	-3.3793	-0.5638	-0.4787	-3.3772	-0.6284	-0.4984	-3.3109	-0.5939	-0.6136	-3.3121	-0.5927	-0.6028
C	1.9429	-1.0390	2.7129	1.9249	-0.9923	2.7518	1.9252	-1.0046	2.7429	1.9373	-1.0075	2.7314
C	1.3188	-2.3139	2.1832	1.3121	-2.2882	2.2629	1.3044	-2.3019	2.2518	1.3105	-2.3028	2.2442
C	-1.0167	-1.9640	2.6942	-1.0343	-1.9530	2.7261	-1.0697	-1.9725	2.6971	-1.0595	-1.9672	2.7047
C	-2.3093	-1.3395	2.2091	-2.3229	-1.3468	2.2078	-2.3486	-1.3550	2.1541	-2.3422	-1.3510	2.1701
C	-1.9786	1.0070	2.6674	-1.9694	1.0082	2.6139	-1.9853	1.0067	2.5899	-1.9878	1.0119	2.5970
C	-1.3379	2.2945	2.1841	-1.3417	2.2945	2.1051	-1.3494	2.3060	2.1135	-1.3478	2.3082	2.1185
C	0.9832	1.9237	2.7941	0.9902	1.9648	2.7201	0.9960	1.9670	2.7289	0.9984	1.9629	2.7283
C	2.2900	1.3128	2.3249	2.2983	1.3395	2.2674	2.3073	1.3456	2.2663	2.3082	1.3435	2.2599
C	-0.3484	-3.1883	0.7055	-0.3190	-3.2103	0.7741	-0.3244	-3.2229	0.7335	-0.3254	-3.2289	0.7429
C	-3.2630	0.3257	0.7473	-3.2706	0.2903	0.7085	-3.2282	0.3164	0.6075	-3.2440	0.3050	0.6291
C	0.4138	3.2420	0.8550	0.4009	3.2684	0.7787	0.4061	3.2856	0.7752	0.4069	3.2806	0.7754
C	3.2793	-0.3025	0.8493	3.2768	-0.3598	0.8615	3.2912	-0.3494	0.8358	3.2879	-0.3409	0.8185
C	3.3712	0.6076	-0.3486	3.4057	0.5100	-0.3637	3.3787	0.4934	-0.4160	3.3604	0.4900	-0.4431
C	4.5785	1.1927	-0.7165	4.6452	0.9918	-0.7709	4.5922	0.9971	-0.8812	4.5738	0.9908	-0.9129
C	4.6377	2.0190	-1.8435	4.7432	1.7886	-1.9175	4.6385	1.7371	-2.0758	4.6226	1.7126	-2.1177
C	3.4558	2.2068	-2.5696	3.5587	2.0611	-2.6138	3.4229	1.9292	-2.7571	3.4083	1.8904	-2.8042
C	2.2868	1.6064	-2.1223	2.3595	1.5534	-2.1336	2.2521	1.4137	-2.2177	2.2367	1.3820	-2.2593
C	-3.4201	-1.0865	-3.5465	-3.3151	-1.1651	-3.5809	-3.2714	-1.1053	-3.7488	-3.2815	-1.0395	-3.7490
C	5.9008	2.6954	-2.2240	6.0492	2.3379	-2.3530	5.9054	2.3172	-2.5841	5.8902	2.2873	-2.6304
C	6.5715	3.5306	-1.3064	6.8760	3.0405	-1.4492	6.7355	3.1082	-1.7494	6.7120	3.0981	-1.8069
C	6.4575	2.5338	-3.5019	6.5066	2.1722	-3.6713	6.3141	2.1266	-3.9231	6.3075	2.0711	-3.9627
C	7.7541	4.1786	-1.6518	8.1088	3.5515	-1.8446	7.9111	3.6857	-2.2324	7.8884	3.6704	-2.2945
C	7.6526	3.1661	-3.8692	7.7464	2.6727	-4.0903	7.4971	2.6922	-4.4233	7.4920	2.6300	-4.4669
C	8.2844	3.9853	-2.9323	8.5332	3.3577	-3.1637	8.2819	3.4732	-3.5680	8.2685	3.4315	-3.6230
C	6.5901	4.5568	0.8385	7.1829	3.9098	0.7425	7.0677	4.1018	0.4144	7.0166	4.1499	0.3339
C	6.3057	1.5096	-5.6463	6.1256	1.2478	-5.8328	5.8440	1.1090	-6.0542	5.8504	1.0135	-6.0768
C	10.0236	4.4968	-4.4714	10.2487	3.6904	-4.7778	9.9008	3.8989	-5.2927	9.8897	3.8380	-5.3500
c1ax	1.3201	-0.6251	3.5162	1.2834	-0.5468	3.5225	1.2762	-0.5575	3.5055	1.2997	-0.5630	3.5055
c1eq	2.9269	-1.2717	3.1590	2.8983	-1.2044	3.2303	2.8900	-1.2273	3.2363	2.9087	-1.2321	3.2112
c2ax	1.9565	-2.7489	1.4021	1.9626	-2.7540	1.5108	1.9540	-2.7624	1.4966	1.9551	-2.7675	1.4868
c2eq	1.2636	-3.0571	2.9993	1.2464	-2.9986	3.1066	1.2488	-3.0154	3.0949	1.2569	-3.0157	3.0881
c3ax	-0.5791	-1.3534	3.4953	-0.6149	-1.3287	3.5259	-0.6590	-1.3482	3.5010	-0.6431	-1.3420	3.5054
c3eq	-1.2355	-2.9568	3.1285	-1.2536	-2.9400	3.1728	-1.3039	-2.9567	3.1455	-1.2904	-2.9505	3.1568
c4ax	-2.7682	-1.9955	1.4610	-2.7665	-2.0213	1.4667	-2.7743	-2.0191	1.3941	-2.7780	-2.0188	1.4184
c4eq	-3.0215	-1.2695	3.0515	-3.0483	-1.2597	3.0370	-3.0965	-1.2768	2.9647	-3.0823	-1.2694	2.9878
c5ax	-1.3835	0.5780	3.4842	-1.3598	0.5951	3.4279	-1.3802	0.5827	3.4011	-1.3874	0.5902	3.4133
c5eq	-2.9757	1.2377	3.0852	-2.9588	1.2448	3.0467	-2.9786	1.2362	3.0198	-2.9819	1.2452	3.0236
c6ax	-1.9311	2.7306	1.3679	-1.9424	2.7107	1.2842	-1.9450	2.7391	1.2993	-1.9449	2.7468	1.3076
c6eq	-1.3369	3.0311	3.0087	-1.3518	3.0425	2.9189	-1.3717	3.0351	2.9456	-1.3629	3.0370	2.9511
c7ax	0.5171	1.2818	3.5525	0.5237	1.3435	3.4948	0.5281	1.3309	3.4903	0.5338	1.3272	3.4925
c7eq	1.1909	2.8964	3.2768	1.1975	2.9476	3.1805	1.2056	2.9397	3.2125	1.2083	2.9361	3.2109
c8ax	2.7824	1.9841	1.6081	2.7835	1.9810	1.5188	2.7786	1.9869	1.5094	2.7800	1.9887	1.5061
c8eq	2.9770	1.2099	3.1840	2.9902	1.2765	3.1266	3.0104	1.2947	3.1185	3.0132	1.2880	3.1102
a1ax	-1.3923	-3.1004	0.3665	-1.3567	-3.1368	0.4124	-1.3565	-3.1341	0.3639	-1.3698	-3.1713	0.4019
a1eq	-0.2236	-4.1697	1.1958	-0.1982	-4.1804	1.2871	-0.2162	-4.2043	1.2289	-0.1829	-4.2061	1.2383
a3ax	1.5025	3.2374	0.6919	1.4911	3.2835	0.6260	1.4940	3.2800	0.6130	1.4973	3.2927	0.6303
a3eq	0.1568	4.1971	1.3451	0.1265	4.2179	1.2700	0.1523	4.2452	1.2600	0.1331	4.2399	1.2499
apax	3.2040	-1.3397	0.4910	3.1660	-1.4026	0.5315	3.1830	-1.3971	0.5214	3.2022	-1.3953	0.5184
apeq	4.2013	-0.2204	1.4513	4.1998	-0.2911	1.4639	4.2268	-0.2671	1.4176	4.2253	-0.2362	1.3940
py3	5.4680	1.0147	-0.1122	5.5319	0.7532	-0.1843	5.5011	0.8204	-0.3073	5.4815	0.8242	-0.3341
py6	1.3431	1.7623	-2.6480	1.4178	1.7693	-2.6420	1.2849	1.5716	-2.6984	1.2756	1.5352	-2.7539
asax	-3.1440	1.3650	0.4108	-3.1412	1.3193	0.3455	-3.0473	1.3399	0.2531	-3.1077	1.3424	0.2943
aseq	-4.1956	0.2652	1.3391	-4.2053	0.2502	1.2982	-4.1945	0.3037	1.1471	-4.2053	0.2518	1.1760
asaxp	-3.7106	-1.5813	-0.2048	-3.7164	-1.6386	-0.2077	-3.6222	-1.6148	-0.3247	-3.6103	-1.6201	-0.3219
aseqp	-4.1849	-0.1294	-1.0978	-4.1702	-0.2064	-1.1418	-4.1270	-0.1882	-1.2416	-4.1359	-0.1932	-1.2252
MeS	-4.3686	-1.3385	-3.0584	-4.2800	-1.4218	-3.1287	-4.2451	-1.3237	-3.2914	-4.2560	-1.2700	-3.2995
MeS_1	-3.3546	-0.0150	-3.7687	-3.2451	-0.0919	-3.7931	-3.1729	-0.0369	-3.9822	-3.1839	0.0345	-3.9556
MeS_2	-3.2923	-1.6851	-4.4566	-3.1531	-1.7566	-4.4901	-3.1282	-1.7195	-4.6480	-3.1359	-1.6313	-4.6628
py5	3.4374	2.8387	-3.4566	3.5650	2.6823	-3.5080	3.3876	2.4972	-3.6856	3.3735	2.4425	-3.7422
ph3	8.2823	4.8356	-0.9651	8.7575	4.1014	-1.1673	8.5520	4.3069	-1.6113	8.5229	4.3067	-1.6821
ph3_1	8.0755	3.0183	-4.8577	8.0872	2.5245	-5.1100	7.7960	2.5232	-5.4526	7.7980	2.4407	-5.4905
Mep	7.6029	4.2155	1.0985	8.1421	3.3975	0.9092	8.0763	3.6833	0.5673	8.0305	3.7517	0.5054
Mep_1	6.6356	5.5804	0.4379	7.3673	4.9399	0.4032	7.1500	5.1346	0.0369	7.0860	5.1737	-0.0701
Mep_2	5.9555	4.5380	1.7306	6.6079	3.9268	1.6742	6.5238	4.1031	1.3676	6.4659	4.1675	1.2831
Mep_3	6.3702	2.4583	-6.1990	6.2571	2.1962	-6.3747	5.8826	2.0519	-6.6245	5.8926	1.9466	-6.6627
Mep_4	7.2984	1.0374	-5.5992	7.0668	0.6785	-5.8451	6.8097	0.5839	-6.1411	6.8162	0.4863	-6.1499
Mep_5	5.6042	0.8375	-6.1511	5.3349	0.6597	-6.3099	5.0452	0.4726	-6.4557	5.0533	0.3709	-6.4718
Meo	9.3426	4.8551	-5.2580	9.5915	4.1623	-5.5236	9.1758	4.2976	-6.0221	9.1625	4.2225	-6.0849
Meo_1	10.9313	5.1092	-4.4665	11.2315	4.1726	-4.8001	10.8393	4.4630	-5.3675	10.8247	4.4067	-5.4319
Meo_2	10.2901	3.4464	-4.6627	10.3588	2.6196	-5.0065	10.0950	2.8353	-5.5105	10.0907	2.7728	-5.5536

Table 3 Best fit parameters of the traceless susceptibility tensor (Å^3 SI units) obtained from the fit of the experimental PCS data measured at 1 Tesla and 27 °C.

	$[Dy.L^4]$	$[DyL^1]$
χ_{xx}	0.052(3)	0.073(5)
χ_{xy}	-0.154(3)	-0.115(4)
χ_{xz}	0.149(3)	0.154(5)
χ_{yy}	0.276(3)	0.239(6)
χ_{yz}	-0.017(3)	-0.022(5)

In Table 3, and in the main text, the best-fit susceptibility tensors are presented as axiality, rhombicity and three Euler angles. We use the following convention for defining axiality, rhombicity and Euler angles of a traceless susceptibility tensor. Diagonalisation of a traceless susceptibility tensor gives eigenvalues and eigenvectors which are labelled to satisfy the relation $|\chi_x|<|\chi_y|<|\chi_z|$. Then axiality is defined as 3/2 χ_z and rhombicity as $(\chi_x- \chi_y)/2$. In such a definition, axiality and rhombicity have the same sign and the limiting value of the ratio of rhombicity to axiality is 1/3 ($0<\chi_{rh}/\chi_{ax}<1/3$).

Table 4 Optimised structures of $\mathbf{[HLnL^1(H_2O)_2]^+}$: a 9-coordinate mono-capped square antiprismatic geometry (SAP).

	Y			Eu		
Ln	0.0000	0.0000	0.0000	0.0000	0.0000	0.0000
S	-5.7698	1.8975	-0.0704	-5.6526	1.9383	-0.2929
O	-1.3517	1.7308	-0.7236	-1.3082	1.8208	-0.7410
O	-2.2454	3.7723	-0.5887	-2.1916	3.8641	-0.5629
O	1.4119	-1.7437	-0.7405	1.4757	-1.7309	-0.7386
O	2.2432	-3.8103	-0.5813	2.3580	-3.7724	-0.5228
O	0.3089	0.0993	-2.6028	0.3290	0.0434	-2.5968
O	-4.9714	2.7620	0.8336	-4.9513	2.8088	0.6828
O	-7.2068	2.2035	-0.2534	-7.0690	2.2320	-0.6103
O	-1.5625	-1.5786	-1.2650	-1.4868	-1.6633	-1.3314
O	3.8374	6.0209	-0.8454	3.5430	6.0794	-0.9252
O	6.4722	2.4485	-2.3446	6.5952	2.6600	-1.9219
O	8.2434	6.9509	-2.3452	7.9716	7.2920	-2.1076
N	2.1461	0.0000	1.5976	2.1386	0.0000	1.6431
N	0.0410	2.0872	1.6464	0.0364	2.1016	1.6709
N	-2.1626	-0.0119	1.7183	-2.1531	-0.0131	1.6502
N	-0.0245	-2.0753	1.6198	-0.0219	-2.0886	1.5862
N	1.9637	1.5370	-0.7425	1.9819	1.4987	-0.7258
N	-5.7009	0.2950	0.4544	-5.6269	0.3354	0.2320
C	2.0872	1.0643	2.6293	2.0555	1.0529	2.6850
C	1.3961	2.3434	2.1785	1.3822	2.3394	2.2319
C	-0.8576	1.8062	2.7858	-0.8902	1.7989	2.7804
C	-2.2249	1.3077	2.3779	-2.2431	1.2990	2.3253
C	-2.0449	-1.0895	2.7203	-2.0713	-1.1080	2.6401
C	-1.3804	-2.3307	2.1546	-1.3946	-2.3456	2.0765
C	0.9158	-1.8325	2.7339	0.8846	-1.8448	2.7285
C	2.2628	-1.3154	2.2684	2.2434	-1.3208	2.3051
C	3.3287	0.1902	0.7360	3.3356	0.2077	0.8047
C	-0.4557	3.2535	0.9003	-0.4290	3.2887	0.9365
C	-1.4487	2.9055	-0.2099	-1.4072	2.9809	-0.1979
C	-3.3385	-0.1958	0.8500	-3.2968	-0.1939	0.7370
C	-4.7091	0.0010	1.5064	-4.6922	0.0256	1.3298
C	0.4114	-3.2411	0.8354	0.4477	-3.2444	0.8052
C	1.4571	-2.9212	-0.2312	1.5320	-2.9046	-0.2188
C	3.1755	1.3670	-0.1892	3.1762	1.3834	-0.1215
C	4.2416	2.2194	-0.4594	4.2105	2.2877	-0.3396
C	4.0597	3.3092	-1.3173	4.0159	3.3712	-1.2047
C	2.7874	3.4842	-1.8763	2.7668	3.4761	-1.8318
C	1.7825	2.5771	-1.5690	1.7921	2.5258	-1.5667
C	-4.9916	1.9093	-1.6624	-4.7305	1.9610	-1.8063
C	5.1708	4.2477	-1.6045	5.0781	4.3778	-1.4354
C	6.3952	3.7852	-2.1134	6.3816	3.9953	-1.7969
C	5.0322	5.6301	-1.3609	4.8062	5.7578	-1.3052
C	7.4564	4.6582	-2.3842	7.3873	4.9402	-2.0373
C	6.0759	6.5161	-1.6138	5.7918	6.7139	-1.5321
C	7.2794	6.0182	-2.1256	7.0747	6.2931	-1.9001
C	3.6763	7.3991	-0.5321	3.2221	7.4597	-0.8075
C	7.6858	1.9334	-2.8788	7.8762	2.2285	-2.3642

C	9.4877	6.5006	-2.8651	9.2797	6.9276	-2.5295
H	-0.5228	-0.2168	-2.9920	-0.4811	-0.3138	-2.9954
H	0.8849	-0.6851	-2.6408	0.9328	-0.7215	-2.5887
H	3.1100	1.3146	2.9635	3.0694	1.2954	3.0505
H	1.5690	0.6544	3.5049	1.5116	0.6356	3.5412
H	1.3431	3.0266	3.0460	1.3205	3.0175	3.1031
H	1.9806	2.8572	1.4072	1.9842	2.8535	1.4740
H	-0.9787	2.7237	3.3906	-1.0328	2.7036	3.3994
H	-0.3782	1.0564	3.4279	-0.4255	1.0380	3.4207
H	-2.8496	1.2664	3.2867	-2.8951	1.2397	3.2133
H	-2.7174	2.0140	1.6938	-2.7202	2.0122	1.6372
H	-3.0376	-1.3733	3.1123	-3.0778	-1.3914	2.9947
H	-1.4781	-0.7063	3.5781	-1.5316	-0.7386	3.5210
H	-1.3422	-3.1105	2.9374	-1.3851	-3.1354	2.8498
H	-1.9925	-2.7309	1.3347	-1.9809	-2.7299	1.2304
H	1.0638	-2.7669	3.3056	1.0188	-2.7798	3.3023
H	0.4632	-1.1008	3.4155	0.4106	-1.1164	3.3989
H	2.9383	-1.2435	3.1387	2.8923	-1.2540	3.1956
H	2.7310	-2.0248	1.5737	2.7358	-2.0229	1.6200
H	3.4435	-0.7127	0.1195	3.4711	-0.6914	0.1867
H	4.2414	0.3066	1.3452	4.2357	0.3364	1.4297
H	0.3924	3.7410	0.3943	0.4365	3.7686	0.4528
H	-0.9087	4.0022	1.5719	-0.8787	4.0357	1.6119
H	-3.3113	-1.1996	0.4090	-3.2568	-1.2023	0.3086
H	-3.2267	0.5257	0.0304	-3.1402	0.5127	-0.0881
H	-0.4558	-3.6315	0.2800	-0.3982	-3.6327	0.2169
H	0.7827	-4.0513	1.4857	0.8007	-4.0589	1.4597
H	5.2050	2.0428	0.0188	5.1553	2.1583	0.1873
H	2.5800	4.3178	-2.5459	2.5528	4.2933	-2.5184
H	0.7845	2.6859	-1.9986	0.8121	2.5820	-2.0441
H	-4.6930	0.7976	2.2623	-4.6991	0.8225	2.0855
H	-5.0556	-0.9174	1.9942	-5.0754	-0.8873	1.8003
H	-6.6545	0.0793	0.7599	-6.5977	0.1293	0.4857
H	-3.9213	1.7028	-1.5440	-3.6653	1.8122	-1.5890
H	-5.4926	1.1640	-2.2901	-5.1385	1.1852	-2.4636
H	-5.1425	2.9232	-2.0554	-4.8911	2.9619	-2.2280
H	-0.8726	-2.1061	-1.7052	-0.7622	-2.1809	-1.7244
H	-2.0634	-2.2455	-0.7692	-2.0359	-2.3396	-0.9036
H	8.3914	4.2834	-2.7882	8.3843	4.6240	-2.3269
H	5.9952	7.5835	-1.4228	5.6051	7.7801	-1.4309
H	4.4159	7.7212	0.2158	3.8352	7.9436	-0.0328
H	3.7655	8.0223	-1.4341	3.3579	7.9792	-1.7677
H	2.6679	7.4959	-0.1168	2.1670	7.4952	-0.5163
H	7.9023	2.3733	-3.8636	8.1045	2.6319	-3.3618
H	8.5281	2.1163	-2.1952	8.6623	2.5241	-1.6538
H	7.5286	0.8551	-2.9858	7.8205	1.1361	-2.4132
H	10.1123	7.3945	-2.9644	9.8244	7.8681	-2.6626
H	9.9705	5.7901	-2.1774	9.7864	6.3146	-1.7690
H	9.3582	6.0313	-3.8520	9.2491	6.3821	-3.4847

References

[1] M. J. Frisch, G. W. Trucks, H. B. Schlegel, G. E. Scuseria, M. A. Robb, J. R. Cheeseman, G. Scalmani, V. Barone, G. A. Petersson, H. Nakatsuji, M. C. X. Li, A. Marenich, J. Bloino, B. G. Janesko, R. Gomperts, B. Mennucci, H. P. Hratchian, J. V. Ortiz, A. F. Izmaylov, J. L. Sonnenberg, D. Williams-Young, F. Ding, F. Lipparini, F. Egidi, J. Goings, B. Peng, A. Petrone, T. Henderson, D. Ranasinghe, V. G. Zakrzewski, N. R. J. Gao, G. Zheng, W. Liang, M. Hada, M. Ehara, K. Toyota, R. Fukuda, J. Hasegawa, M. Ishida, T. Nakajima, Y. Honda, O. Kitao, H. Nakai, T. Vreven, K. Throssell, J. A. M. Jr., J. E. Peralta, F. Ogliaro, M. Bearpark, J. J. Heyd, E. Brothers, K. N. Kudin, V. N. Staroverov, T. Keith, R. Kobayashi, J. Normand, K. Raghavachari, A. Rendell, J. C. Burant, S. S. Iyengar, J. Tomasi, M. Cossi, J. M. Millam, M. Klene, C. Adamo, R. Cammi, J. W. Ochterski, R. L. Martin, K. Morokuma, O. Farkas, J. B. Foresman, D. J. Fox, Revision D.01 ed., Gaussian, Inc., Wallingford CT, **2016**.

[2] Y. Zhao, D. G. Truhlar, *Theoretical Chemistry Accounts: Theory, Computation, and Modeling (Theoretica Chimica Acta)* **2008**, *120*, 215-241.

[3] T. H. Dunning Jr, *J. Chem. Phys.* **1989**, *90*, 1007-1023.

[4] aK. L. Schuchardt, B. T. Didier, T. Elsethagen, L. Sun, V. Gurumoorthi, J. Chase, J. Li, T. L. Windus, *Journal of chemical information and modeling* **2007**, *47*, 1045-1052; bM. Dolg, H. Stoll, H. Preuss, R. M. Pitzer, *The Journal of Physical Chemistry* **1993**, *97*, 5852-5859.

[5] A. V. Marenich, C. J. Cramer, D. G. Truhlar, *J. Phys. Chem. B* **2009**, *113*, 6378-6396.
[6] S. Grimme, J. Antony, S. Ehrlich, H. Krieg, *J. Chem. Phys.* **2010**, *132*, 154104.
[7] H. J. Hogben, M. Krzystyniak, G. T. P. Charnock, P. J. Hore, I. Kuprov, *J Magn Reson* **2011**, *208*, 179-194.
[8] Z. Kovacs, A. D. Sherry, *J. Chem. Soc. Chem. Commun.* **1995**, *0*, 185-186.
[9] J. Chen, V. Palani, T. R. Hoye, *Journal of American Chemical Society*, **2016**, *138*, 4318-4321.
[10] E. J. New, D. Parker and R. D. Peacock, *Dalton Trans.*, 2009, 672–679,and reference 14 in the main text
[11] E. A. Suturina, K. Mason, C. F. G. C. Geraldes, I. Kuprov, D. Parker, *Angewandte Chemie International Edition* **2017**, *56*, 12215-12218.
